# Hypoxia-Induced Adaptations of miRNomes and Proteomes in Melanoma Cells and Their Secreted Extracellular Vesicles

**DOI:** 10.3390/cancers12030692

**Published:** 2020-03-14

**Authors:** Geoffroy Walbrecq, Odile Lecha, Anthoula Gaigneaux, Miriam R. Fougeras, Demetra Philippidou, Christiane Margue, Milène Tetsi Nomigni, François Bernardin, Gunnar Dittmar, Iris Behrmann, Stephanie Kreis

**Affiliations:** 1 Department of Life Sciences and Medicine, University of Luxembourg, 6, avenue du Swing, L-4367 Belvaux, Luxembourg; geoffroy.walbrecq@uni.lu (G.W.); odile.lecha@uni.lu (O.L.); anthoula.gaigneaux@uni.lu (A.G.); demetra.philippidou@uni.lu (D.P.); christiane.margue@uni.lu (C.M.); milene.tetsi@uni.lu (M.T.N.); gunnar.dittmar@lih.lu (G.D.); iris.behrmann@uni.lu (I.B.); 2 Proteomics of Cellular Signaling, Quantitative Biology Unit, Luxembourg Institute of Health, 1A-B, rue Thomas Edison, L-1445 Strassen, Luxembourg; miriam.fougeras@lih.lu (M.R.F.);; 3 Doctoral School in Science and Engineering (DSSE), Faculty of Science, Technology and Medicine, University of Luxembourg, 2 avenue de l’Université, L-4365 Esch-sur-Alzette, Luxembourg

**Keywords:** melanoma, hypoxia, extracellular vesicles, proteome, miRNome

## Abstract

Reduced levels of intratumoural oxygen are associated with hypoxia-induced pro-oncogenic events such as invasion, metabolic reprogramming, epithelial–mesenchymal transition, metastasis and resistance to therapy, all favouring cancer progression. Small extracellular vesicles (EV) shuttle various cargos (proteins, miRNAs, DNA and others). Tumour-derived EVs can be taken up by neighbouring or distant cells in the tumour microenvironment, thus facilitating intercellular communication. The quantity of extracellular vesicle secretion and their composition can vary with changing microenvironmental conditions and disease states. Here, we investigated in melanoma cells the influence of hypoxia on the content and number of secreted EVs. Whole miRNome and proteome profiling revealed distinct expression patterns in normoxic or hypoxic growth conditions. Apart from the well-known miR-210, we identified miR-1290 as a novel hypoxia-associated microRNA, which was highly abundant in hypoxic EVs. On the other hand, miR-23a-5p and -23b-5p were consistently downregulated in hypoxic conditions, while the protein levels of the miR-23a/b-5p-predicted target *IPO11* were concomitantly upregulated. Furthermore, hypoxic melanoma EVs exhibit a signature consisting of six proteins (AKR7A2, DDX39B, EIF3C, FARSA, PRMT5, VARS), which were significantly associated with a poor prognosis for melanoma patients, indicating that proteins and/or miRNAs secreted by cancer cells may be exploited as biomarkers.

## 1. Introduction

Melanoma is the most aggressive form of skin cancer with an annual increase of 0.6% estimated among adults over 50 years [1]. Melanoma develops from melanocytes, which originate from neural crest-derived cells. Primary transformed melanocytes first form a melanoma in situ. In order to expand, the melanoma in situ will “create” an immunosuppressive environment, which allows the tumour cells to invade the lower dermis as well as blood or lymphatic vessels paving the way for metastatic disease [2]. With increasing size of the primary or metastatic tumour, cells in the centre experience a lack of oxygen, which has been shown to induce a number of hypoxia-inducible genes involved in the adaptation of cells to a lower oxygen supply [3]. Hypoxia has been demonstrated to be an aggravating factor in tumour development facilitating metastasis [4], e.g., by promoting lymph node metastasis in melanoma [5]. Furthermore, hypoxia induces distinct changes in the tumour microenvironment (TME) by increasing levels of glycolysis, by inducing autophagy and by maintaining a stem cell-like character of the cells [3,4]. Many of the hypoxia-induced changes to the TME are triggered by prior transfer of secreted molecules via EVs [6].

Small extracellular vesicles (EVs) are vesicles (30–200 nm) involved in intercellular communication, which carry bioactive molecules such as proteins, DNA, mRNAs, non-coding RNAs such as miRNAs and long non-coding RNAs, as well as metabolites [7]. Cancer cells produce more EVs than normal cells [8]. Recent evidence has shown their involvement in tumour initiation, progression, metastasis, drug resistance, the escape of immune surveillance and in shaping of the TME [7]. Likewise, several studies have described the influence of hypoxia on EVs of different cancer types: hypoxic EVs stimulate cancer angiogenesis via Wnt4, miR-135b and miR-23a [9,10], and invasion and metastasis through a processes mediated by RAB22A [11]. In addition, Li et al. also demonstrated that hypoxic EVs from oral squamous cell carcinoma cells induce increased migration and invasion in a process involving miR-21 [12]. The effect of hypoxic EVs on melanoma cells and cells from the melanoma TME is, however, still not well characterised. 

Here, we lowered the oxygen concentration applied to melanoma cells to 1% and isolated EVs of four melanoma cell lines (two carrying the BRAF V600E mutation (A375, 501Mel) and two carrying an NRAS mutation (MelJuso, IPC298)); control cells were kept under normoxic conditions. We profiled proteomes and miRNomes of normoxic and hypoxic melanoma EVs (nEVs, hEVs) and their corresponding whole cell lysates (WCLs). Overall, hypoxic melanoma cells released more EVs than normoxic cells, indicating that cells in the hypoxic core of a tumour would contribute more to the secretome than normoxic cells of the periphery. We also show that hEVs promote invasion but not migration or proliferation of A375 melanoma cells. Altogether, hypoxic melanoma EVs present specific protein and miRNA signatures, which could be further explored in order to determine their role in the context of the melanoma microenvironment and as potential biomarkers to predict disease prognosis or response to therapy.

## 2. Results

**Figure 1:**
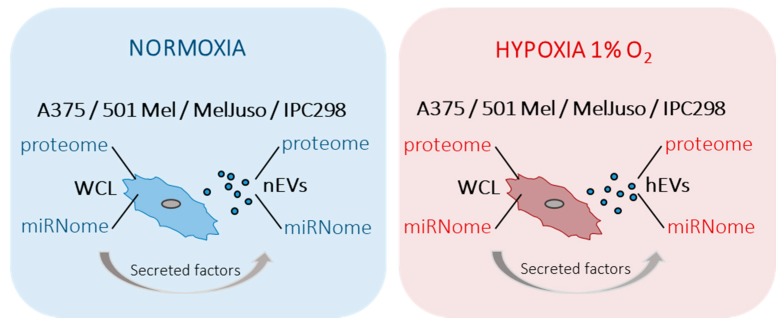
Schematic overview of the study design. Four different melanoma cell lines (A375, 501Mel, MelJuso and IPC298) were cultured under normoxic (blue) or hypoxic (red) conditions. Proteomes and miRNomes of the cells and of the respective small extracellular vesicles (EVs) were characterised by mass spectrometry and qPCR arrays and miRNA microarrays, respectively. WCL: whole cell lysate, nEVs: normoxic small extracellular vesicles, hEVs: hypoxic small extracellular vesicles.

In order to get insights into the various molecular messengers operating in a hypoxic microenvironment in melanoma, the proteomes and miRNomes of whole cell lysates (WCLs) and EVs isolated from supernatants of cells cultured under normoxia or hypoxia, were investigated (Figure 1). Including miRNAs in this study further allowed us to determine the possible influence of post-transcriptional gene regulation ultimately resulting in altered protein levels inside the cell as well as in secreted vesicles. The content of EVs is known to reflect the cell of origin and can partake in intercellular communication. Thus, EVs shuttle different signals between cells grown under different conditions [13]. 

### 2.1. Characterisation of Small Extracellular Vesicles

**Figure 2:**
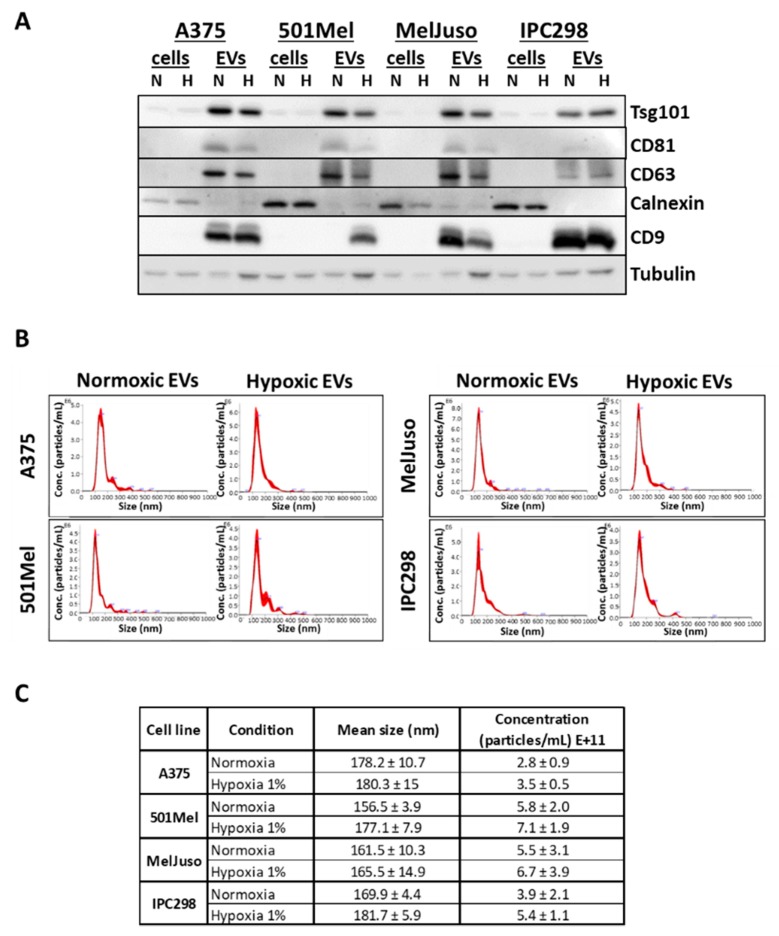
Characterisation of normoxic (N) and hypoxic (H) melanoma small extracellular vesicles (EVs) and whole cell lysates (cells). (**A**) Western blot of A375, 501Mel, MelJuso and IPC298 cells and EVs under normoxia or hypoxia, probed with antibodies against the EV markers Tsg101, CD81, CD63, CD9 and the endoplasmic reticulum marker Calnexin, as well as Tubulin. (**B**) Nanosight measurements of EVs from A375, MelJuso, 501Mel, IPC298 cells. (**C**) Size and concentration of EVs secreted under normoxia or hypoxia for the four melanoma cell lines as measured by Nanosight. Three biological replicates were used for each condition.

First, we determined the quality, purity and number of isolated EVs. nEVs and hEVs were isolated by ultracentrifugation from cell supernatants of four melanoma cell lines A375, 501Mel, MelJuso and IPC298. The purity of isolated EVs was assessed by Western blot analysis to confirm the expression of EV markers (CD9, CD63, CD81 and Tsg101). All EV markers were enriched in EV preparations (with the exception of CD9, which was inconsistently detected in 501Mel EVs), while Calnexin, absent in EVs and a marker of the endoplasmic reticulum, was only present in cell lysates (Figure 2A). Expression of the hypoxia marker HIF-1α was verified in hypoxic WCLs (Figure S1). The quality, concentration and size of the EV preparations was further analysed using nanoparticle tracking analysis (NTA). The EV samples show a main single peak at +/-170 nm (Figure 2B) and appropriate size and concentration ranges (Figure 2C). As previously reported, the secretion of EVs was slightly increased under hypoxia [8,12]. 

### 2.2. Influence of Hypoxia on the Proteome of Melanoma Cells and Their Secreted Vesicles

**Figure 3:**
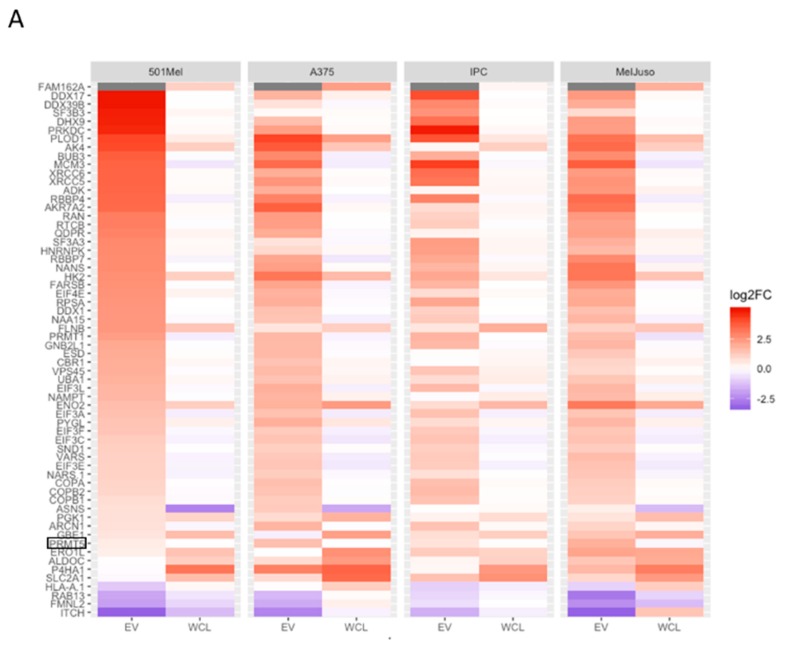

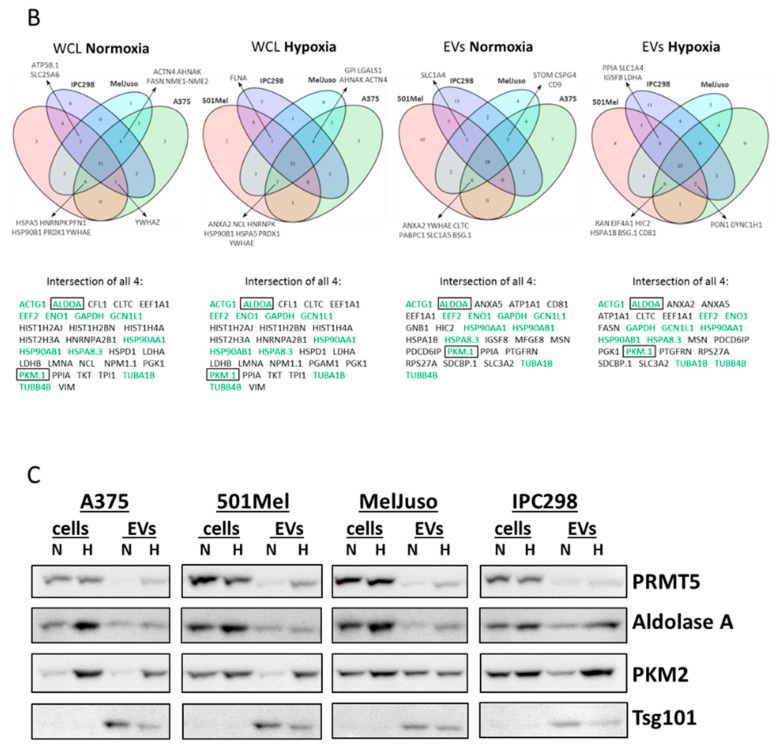
Proteomic analysis of nEVs and hEVs, normoxic and hypoxic whole cells lysates (WCLs) of melanoma cells. (**A**) Heatmap showing the proteins differentially expressed between normoxia and hypoxia in at least two cell lines, either in EVs or in WCLs, and not imputed in this compartment (either EVs or WCLs). Using these criteria, 49 proteins were identified in EVs, and 14 proteins were identified in WCL. Colour shows normalised expression. Three biological replicates were used for the melanoma cell lines (A375, 501Mel, IPC298), and only two biological replicates for the melanoma cell line MelJuso due to technical problems. (**B**) Venn diagrams showing the intersections of the top 50 expressed proteins for the four melanoma cell lines in WCL or EV under normoxia or hypoxia. The common proteins for the four melanoma cell lines are indicated below the Venn diagrams. Common proteins for all groups are written in green. Black boxes indicate proteins further validated by Western blot. (**C**) Expression analysis of ALDOA, PKM2, PRMT5, Tsg101 (EV marker) in normoxic or hypoxic WCL and nEVs or hEVs of four melanoma cell lines (A375, MelJuso, IPC298, 501Mel) by Western blot.

The proteomes of WCLs and EVs of normoxic and hypoxic A375, 501Mel, MelJuso and IPC298 melanoma cells were characterised by mass spectrometry (Spreadsheet S1). The total number of proteins (≈3000) identified for WCL under normoxia was smaller compared to WCL under hypoxia (*p*-value = 3.84×10 ^−4^) (Figure S2). Overall, around 2000 proteins were detected in matching EVs and the number of proteins identified in hEVs was higher compared to nEVs (*p*-value = 7.62×10 ^−3^) (Figure S2). In addition, we found that at least 81 proteins in each of the eight melanoma EV samples were among the top 100 identified EV markers as listed in Exocarta (exocarta.org) (Figure S3), which further confirmed the quality of our EV isolation. The heatmap in Figure S4A shows the expression for all identified proteins without hierarchical clustering. The heatmap depicting fold changes of all differentially expressed proteins upon hypoxia reveals that hypoxia has a bigger impact on EVs than on WCLs (Figure S4B). Volcano plots of proteins differentially expressed under hypoxia indicate a stronger effect on the upregulated fraction with only few proteins consistently downregulated (Figure S5). The heatmap in Figure 3A shows a selection of proteins, which were differentially expressed between normoxia and hypoxia in at least two cell lines, either in EVs or WCLs, and which were not imputed (see Section 4 for imputation). We found more proteins matching those criteria in EVs (49 proteins) as compared to WCLs (14 proteins). Proteins such as FAM162A, ENO2, P4HA1 were increased in hypoxic cells while helicases such as DDX17, DDX39B and DHX9 were selectively enriched in hypoxic EVs. Overall, in the top expressed proteins, 12 proteins were shared in all samples (Figure 3B) (marked in green), among them, four are involved in glycolysis (ALDOA, ENO1, GAPDH, PKM.1 (as known as PKM)). Between 25 and 31 proteins were common among the top expressed proteins for the four groups of samples (nWCL, hWCL, nEVs and hEVs) indicating that among the highly expressed proteins there was little regulation or difference inside the cell compared to the EVs and with or without application of hypoxia (Figure 3B). Several of the most abundant proteins were well represented in almost all compartments (PKM.1, ENO1, ALDOA) while some EV markers, as defined by Exocarta.org, were predominantly found in EV samples (ACTG1 (in 6/8 samples), ALDOA (7/8), ANXA5 (4/8), EEF2 (4/8), PGK1 (3/8). We also found that melanoma EVs contained several melanoma-specific proteins (seven out of 10 melanoma-specific proteins), whose expression was not influenced by hypoxia (data not shown). 

Next, we validated the differential expression of three proteins identified by proteomics analysis using Western blots (Figure 3C). ALDOA (Aldolase A), PKM2 (Pyruvate kinase isozymes M2), two glycolytic enzymes, which are among the highly expressed proteins in all compartments (Figure 3B), and PRMT5 (Protein arginine methyltransferase 5), which is upregulated in hEVs (Figure 3A). Of note, the gene *PKM.1* can give rise to many isoforms but only PKM2 is expressed in tumour cells where it alters glucose metabolism [14]; thus, we selected PKM2 for the validation of *PKM.1*. The levels of protein expression were concordant with those found in proteomics analysis.

**Figure 4:**
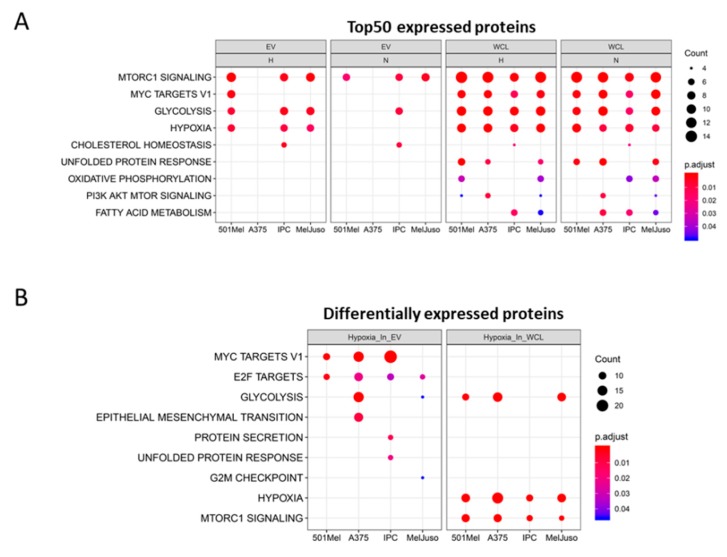
Graph showing enriched hallmarks (**A**) associated with the top 50 expressed proteins in nEVs, hEVs, normoxic or hypoxic WCL and (**B**) associated with differentially expressed proteins. Count is the number of genes identified per hallmark for the indicated compartment. Colour shows the adjusted *p*-value.

To get insights into the signalling pathways and biological processes related to the top 50 proteins of each compartment (WCL or EV), we performed a functional enrichment analysis using hallmark gene sets (Figure 4) and Gene Ontology (GO) “Biological Processes” (Figure S6) on top expressed proteins. A hallmark is a refined gene set characterised by overlapping gene sets, which have common biological themes [15]. We used hallmark gene set analysis as it has fewer overlapping gene sets and thus, it has less redundancy in the results compared to the GO “Biological Process” analysis. Interestingly, the hallmark “MTORC1 signalling” was enriched in almost all conditions (except in A375 EVs), while the hallmark “hypoxia” was enriched in most of the hypoxic conditions, but strikingly also for all WCLs under normoxia (Figure 4A). Proteins of the “unfolded protein response” (UPR) were exclusively found inside the cells but not in EVs, indicating that the proteins of UPR are not exported to EVs. However, nEVs from bladder cancer cells can induce UPR of non-malignant cells and hEVs can transfer miRNAs potentially triggering UPR [16,17]. Moreover, the GO term “viral life cycle” was strongly enriched within the top expressed proteins in EVs (Figure S6A), suggesting a link to the common origin of EVs and viruses. Many viral envelopes are formed using the endosomal system, which is also the origin of EVs, hence the similar protein contents (e.g., ANXA2, CD81) [18]. When taking differentially expressed proteins under hypoxia into account, the enrichment analysis highlighted “Myc targets” and “E2F targets” in most hEVs (Figure 4B). In WCLs, the hallmarks “hypoxia” and “mTORC1 signalling” were enriched under hypoxic conditions, confirming the involvement of the mTOR signalling pathway in responses to lower oxygen levels [19]. Several biological processes related to RNA metabolism were enriched in the hypoxic EV fraction, suggesting that proteins involved in RNA metabolism are shuttled out of the cell when oxygen drops while “amino acid metabolism” and “response to oxygen level” was confined to WCL (Figure S6B). 

**Figure 5:**
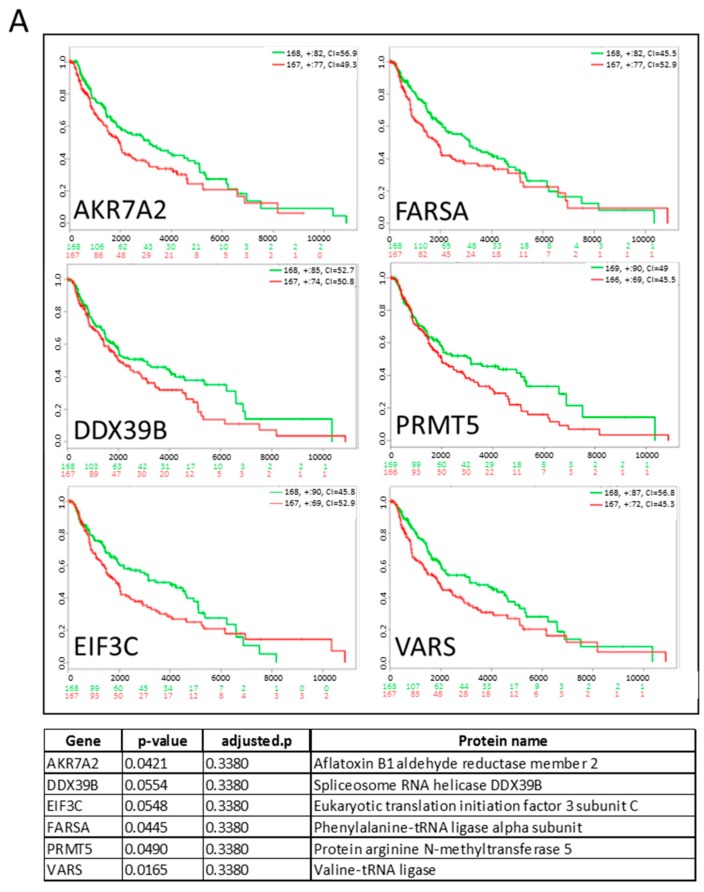

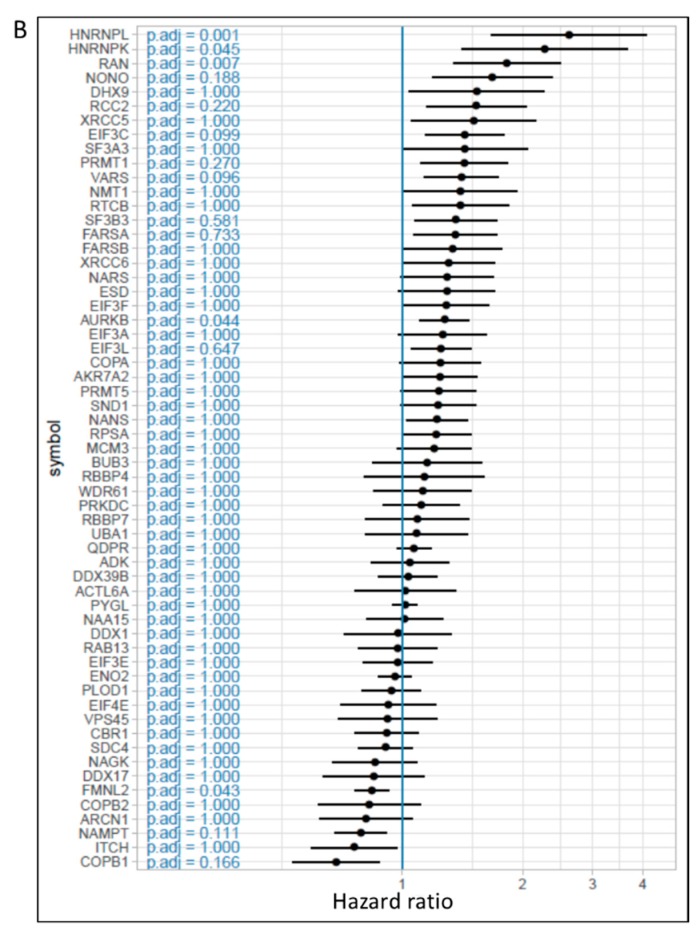
(**A**) Kaplan–Meyer curves of the survival of melanoma patients expressing high or low amounts of the selected genes (dataset: SKCM-TCGA Skin Cutaneous Melanoma July 2016). The table indicates the *p*-value, adjusted *p*-value and the function of the selected genes from Table S1. Low and high expression groups are marked in green and red, respectively. The total patient number for each group is indicated in the top right corner and the number of censoring samples are shown with +. The concordance index (CI) per curve is presented. On the y axis, the probability is represented; on the x axis, the time in days (black) is shown and the number of samples for low (green) and high (red) expression groups who are not presenting the event at the matching time. (**B**) Forest plot representing hazard ratio and confidence intervals from Cox models for the selected genes (dataset: SKCM-TCGA Skin Cutaneous Melanoma July 2016). Models accounting for stage group effect were run separately on each gene and *p*-values were adjusted by false discovery rate (also see Section 4).

To further analyse the influence of proteins in hEVs, we generated a list of differentially expressed proteins under hypoxia in at least two of the four melanoma cell lines and which were imputed less than three times (Table S1). We found 60 proteins, most of which were highly expressed in hEVs, with only six proteins downregulated in hEVs compared to nEVs (AURKB, BUB3, FMNL2, ITCH, RAB13, SDC4). We next evaluated if among the list of differentially expressed proteins in hEVs, some were correlated with a poorer prognosis for melanoma patients. Among them, six (AKR7A2, DDX39B, EIF3C, FARSA, PRMT5, VARS) were upregulated in hEVs compared to nEVs and were significantly (*p* ≤ 0.055) associated with a poor outcome (Figure 5A). This result suggests that some of the hypoxia-induced proteins could be suitable EV biomarkers to predict survival or progression of disease. However, the adjusted *p*-values did not reach significance. Using a Cox regression model, we correlated the gene expression of the 60 proteins differentially expressed in hEVs with survival and tumour stage. The results are shown on the forest plot (Figure 5B) and indicate that three proteins upregulated in hEVs were significantly associated with a poor survival (HNRNPL, HNRNPK, RAN) with *p*-value ≤0.05 while FMNL2 was associated with a better prognosis. Finally, we analysed if the differentially expressed proteins under hypoxia match proteins commonly known to be induced by hypoxia (as described in literature). We generated a heatmap of the “hypoxia signature proteins” and found that they were also strongly upregulated in our data set (Figure S7). Among these 24 proteins, nine are directly involved in glucose metabolism.

### 2.3. Profiling of the miRNome in Melanoma Cells and Their EVs Under Normoxia and Hypoxia

**Figure 6:**
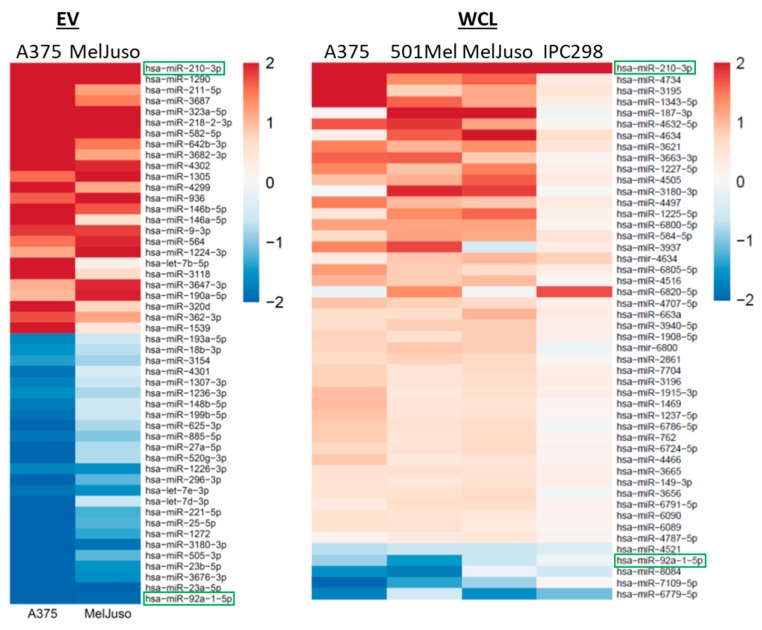
miRNA profiling of melanoma EVs and WCLs. Heatmap showing the top 25 miRNAs differentially up- (red) or downregulated (blue) in A375 and MelJuso hEVs compared to nEVs (left) and regulated miRNAs in hWCLs compared to nWCLs in all four cell lines (right). Colours indicate the fold change in expression relative to normoxia (log2 scale). miRNAs found in both EVs and WCLs are framed in green.

As RNA amounts extracted from EVs are limiting, the miRNomes of nEVs and hEVs were analysed by highly sensitive qPCR arrays for a BRAF mutant (A375) and for an NRAS mutant melanoma cell line (MelJuso), while analysis of the miRNomes of WCLs was performed by miRNA microarrays. MiRNAs differentially regulated under hypoxia in EVs and WCLs are shown in Figure 6. MiR-210, which is well known to be induced by hypoxia, was found upregulated in both hEVs and hWCLs. A previous study has reported miR-23a-5p to be upregulated in hEVs of lung cancer cells [10]. However, our results show that miR-23a-5p is downregulated in melanoma hEVs. MiR-211 was upregulated in hEVs but this result could not be confirmed by individual qPCRs (data not shown). MiR-92a-1-5p levels were reduced under hypoxia in both EVs and WCLs, in line with a previous report showing also decreased expression in WCLs from patient-derived melanoma cells under hypoxia [20]. MiR-92a-1-5p is also downregulated in BRAF inhibitor-resistant melanoma cell lines [21] and one of its targets, S100A9 could play a role in resistance to BRAF inhibitors [22].

We validated by individual qPCRs seven EV miRNAs for all four melanoma cell lines (A375, 501Mel, MelJuso and IPC298), i.e., miRNAs which were found either upregulated (miR-210, miR-1290, miR-323a-5p) or downregulated (let-7d-3p, miR-23b-5p, miR-708-5p, miR-23a-5p) under hypoxia (Table S2). Apart from the previously described miR-210 [23], we consistently saw miR-1290 to be upregulated under hypoxia in EVs but not in WCLs. miR-1290 has previously been found in EVs and has been implicated in oncogenic pathways in gastric and other solid cancers [24,25,26] but has so far not been connected to hypoxic responses. The highest scoring predicted target of miR-1290 is HIGD2A, the HIG1 hypoxia inducible domain family, member 2A (Targetscan.org). 

Next, we searched for proteins differentially expressed in hEVs, which would also be predicted targets of our selected miRNAs miR-1290, miR-23a-5p and miR-23b-5p. We found two proteins, IPO11 (Importin11) and FXR2 (FMR1 autosomal homolog 2), the expression of which was inversely correlated with their targeting miRNAs in A375 cells (Figure S8). IPO11, a predicted target of miR-23a-5p and miR-23b-5p, was upregulated in hEVs. FXR2 is downregulated in hEVs and is a predicted target of miR-1290. In order to validate those predicted targets, we transfected four melanoma cell lines with mimics for either miR1290, miR-23a-5p or miR-23b-5p followed by expression analysis of FXR2 and IPO11 by qPCR and Western blot.

The miR-1290 mimic decreased the expression of FXR2 mRNA in melanoma cells after 48h (Figure S9A) and it induced a reduction of protein level in MelJuso cells (Figure S9B). The miR-23a-5p mimic reduced IPO11 mRNA levels in two of four cell lines after 48h (MelJuso and A375) (Figure S9C) while it did not affect protein levels (Figure S9D). The miR-23b-5p mimic had more pronounced effects in all cell lines after 48 and 72h (except 501Mel) (Figure S9C). Reduction of protein levels following miR-23b-5p mimic transfection was observed after 72h in 501Mel and MelJuso cells but not in A375 and IPC298 cells (Figure S9D). Altogether, FXR2 likely represents a direct target of the newly described hypoxia-regulated miR-1290, and IPO11 is a likely miR-23b-5p target gene. Among the differentially expressed miRNAs in EVs and WCLs, only miR-1290 was exclusively seen in hypoxic EVs but not in WCLs. It has previously been suggested that miRNAs with a specific export sequence would preferentially be sorted into EVs [27]. Indeed, the C/UCCU/G sequence motif is present in the miR-1290 sequence, suggesting this miRNA to be prone for export, triggered by hypoxia. 

### 2.4. Influence of nEVs and hEVs on proliferation, invasion and migration

**Figure 7:**
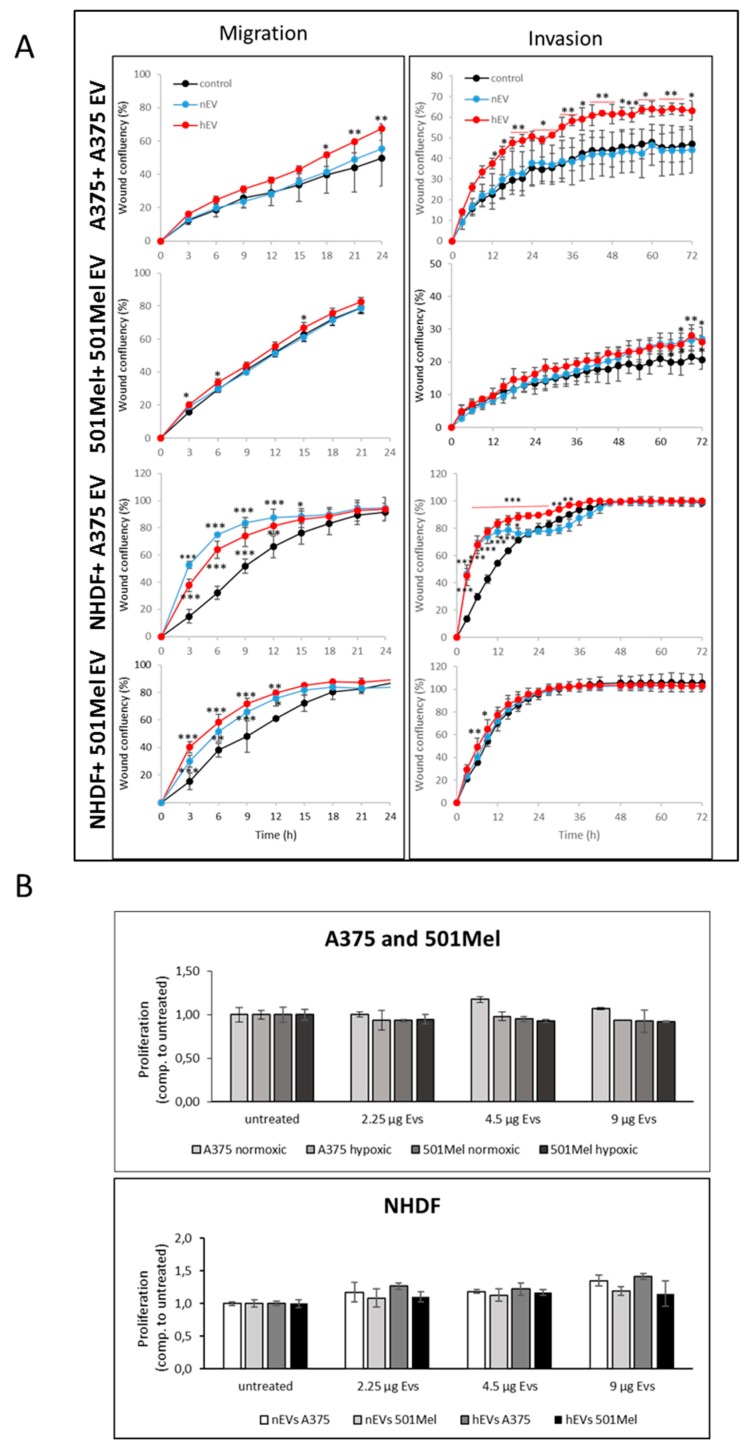
(**A**) Invasion-migration assays. Melanoma nEVs and hEVs from A375 or 501Mel cells were added to their respective cells of origin or to NHDFs. Migration was monitored for 24h and invasion for 72h. * *p* < 0.05, ** *p* < 0.01, *** *p* < 0.001, compared to control treatment. (**B**) Proliferation assays. Melanoma nEVs and hEVs (A375 or 501Mel) were added to melanoma cells (A375 or 501Mel) or to NHDFs. Proliferation was monitored 48 h after EV addition.

Hypoxia has been shown to induce invasion of melanoma cells [28] and another study has discussed that hypoxic EVs induce migration and invasion of naïve tumour cells [29]. Based on these results, we investigated the effect of nEVs and hEVs on the migration and invasion of melanoma cells and normal human dermal fibroblasts (NHDFs). EVs prepared from both normoxic and hypoxic cell supernatants strongly enhanced migration of NHDFs at early time points (i.e., during the first 12 h) (Figure 7A). However, EVs had no effect on migration of 501Mel cells, and only hypoxic EVs slightly increased migration of A375 cells at later time points (i.e., after 18 h). Moreover, A375 hEVs also strongly increased invasion of A375 cells; this effect (more than 30%) starts to be visible after 12h. EVs from A375 cells but not from 501Mel cells also increased invasion of NHDFs at early time points (i.e., during the first day of treatment). Importantly, EVs isolated from normoxic or hypoxic cells did not alter proliferation of melanoma cells or NHDFs (Figure 7B). Taken together, although the miRNA and protein contents of hEVs differs from nEVs and from the corresponding WCL, this does not seem to modulate growth of melanoma cells or of fibroblasts. The effect of EVs on migration and invasion is variable, depending on the cellular source of the EVs, the target cells, as well as on the treatment conditions (i.e., whether EVs were derived from cells cultivated under hypoxia or normoxia).

### 2.5. Influence of Hypoxia on the EV Uptake

Some proteins such as syndecan 4, ERK1/2 and Hsp27 have been proposed to be involved in EV uptake [30,31,32] and are also upregulated or activated under hypoxia [33,34,35]. We thus hypothesised that hypoxia could lead to an increased EV uptake. We compared the uptake of PKH67-labelled nEV by A375, 501Mel and NHDFs under normoxia or hypoxia, by confocal microscopy or with a Cytation 5 plate reader (Biotek) but could not score significant differences employing two independent methods of spot counting (manual counting of EVs in confocal microscopy pictures or using an automatic counting software on Cytation 5 pictures) (Figure S10).

## 3. Discussion

Hypoxia, commonly found in the core of solid tumours, is a major factor influencing melanoma progression, growth [36], invasiveness [28] and resistance to therapy [37]. Extracellular vesicles transport proteins and non-coding RNAs between cells and as such they are important vehicles in intercellular communication [7]. EVs secreted from hypoxic tumour cells have been shown to operate as signalling platforms, which promote tumour angiogenesis, migration, invasion, and the suppression of the immune response [38,39]. Several studies have investigated the role of EVs secreted from normoxic melanoma cells and have shown that such EVs are involved in therapy resistance [40,41] as well as in metastasis by colonising the lymph nodes [42] or by educating the pro-metastatic phenotype through the receptor tyrosine kinase MET [43]. In addition, melanoma EVs contain pro-angiogenic factors, suppress the immune response [44] and stimulate epithelial–mesenchymal transition in primary melanocytes [45]. Much less is known about the influence of hypoxia on the content of melanoma EVs and whether hypoxia would alter the functional impact such hEVs would have on surrounding cells and the tumour microenvironment. 

Here, we studied the effect of hypoxia on the EV and cell content of four melanoma cell lines, two carrying a BRAF V600E mutation and two carrying an NRAS mutation, using qPCR arrays (EVs), miRNA microarrays (WCLs) and mass spectrometry. We confirmed that hypoxia slightly enhances the EV release as was described before for other types of cancers [8,12]. The fact that the EVs number increases with hypoxia might have an influence on the tumour microenvironment, even if the content would stay similar by merely secreting more of a certain miRNA or protein.

Overall more proteins were detected in hypoxic (≈2200) versus normoxic EVs (≈2000). Hypoxic melanoma EVs carried a hypoxic signature consisting of six proteins (AKR7A2, DDX39B, EIF3C, FARSA, PRMT5, VARS) which were significantly associated with a poor prognosis for melanoma patients. EIF3C promotes proliferation, migration and invasion of prostate [46], ovarian and hepatocellular carcinoma cells [47,48] and was associated with resistance to erlotinib in lung cancer [49]. PRMT5 (Protein arginine methyltransferase 5) is upregulated under hypoxia [50] and has many roles in cancer [51]. Recently, PRMT5 was shown to be involved in drug resistance against CDK4/6 inhibitors in melanoma [52]. Using a Cox regression model, we estimated the survival in relation to protein expression and taking tumour stages into account. We found three proteins (HNRNPL, HNRNPK, RAN) upregulated in hEVs, which were associated with decreased survival. Hypoxia has been shown to induce the transport of HNRNPL from the nucleus to the cytoplasm where it can stabilise VEGFA [53] and HNRNPK has been reported to be involved in the tumorigenesis of lung and gastric cancer cells [54,55] while RAN was found to play an important role in the nuclear-cytoplasmic transport of proteins under hypoxia [56].

Some proteins were common to almost all compartments (ALDOA, ENO1, PKM2). ALDOA promotes cancer cell invasion but also glycolysis [57]. While, ENO1, like PKM2, promotes the Warburg effect, but also cancer invasion [58]. In addition, ENO1 and ALDOA genes have both hypoxia-responsive elements for HIF1α [59] and PKM2 is upregulated under hypoxia promoting HIF1α transactivation [60]. Interestingly, PKM2 inhibitors (e.g., shikonin), which target oxidative phosphorylation, have been shown to improve the therapeutic effects of combined BRAF/MEK inhibitor [61]. In this context, hepatic stellate cells under hypoxia increase expression of GLUT1 and PKM2, which were enriched in our hEVs. The delivery of GLUT1 and PKM2 by hEVs could thus lead to a glycolysis increase in the recipient cells [62]. Another study by Shu et al. demonstrated that melanoma EVs induce a metabolic reprogramming of fibroblasts by transferring miR-210 and miR-255 [63]. Since PKM2 and miR-210 are upregulated in melanoma hEVs, transfer of hEVs could cause a reprogramming towards a glycolytic metabolism of the recipient cells. Altogether, these findings suggest that vesicles secreted by tumour cells, especially from the hypoxic core of the tumours, could influence the level and state of glycolysis of cells present in the tumour microenvironment. 

An enrichment of proteins involved in the GO biological processes “RNA splicing” and “regulation of mRNA splicing via spliceosome” was also present for the top differentially expressed proteins in hEVs of 501Mel and IPC298 cells. A recent study has shown that the transfer of splicing factors through EV transfer promotes malignancy of glioblastoma cells [64]. In addition, splicing factors are also present in EVs derived from apoptosis-resistant leukaemia cells [65] and derived from RAS transformed cells [66]. RNA Binding Motif Protein X-Linked (RBMX) and Heterogeneous Nuclear Ribonucleoprotein L (HNRNPL) belong to the GO biological process “RNA splicing” and were enriched in hEVs (RBMX: hEVs IPC298; HNRNPL: hEVs 501Mel, hEVs IPC298). lncRNAs containing RBMX binding motifs were enriched in prostate EVs and those lncRNAs could also influence the miRNA loading in EVs by acting as miRNAs sponges [67]. This could partially explain why the miRNA content in hEVs is different to the miRNA content in nEVs. 

The miRNome analysis of melanoma EVs allowed to identify up- and downregulated miRNAs in hypoxic EVs such as miR-210 and miR-1290 (up) and miR-23a-5p and miR-23b-5-p (down) which were validated in all four cell lines (Table S2). miRNAs have been shown to have important roles in the progression of melanoma [68]. Among the top-regulated miRNAs was miR-210, known to respond to hypoxia and with many documented roles in cancer [23]. Here, we identified a new hypoxia-associated miRNA, miR-1290, which promotes cell migration and invasion of cancer cells [69,70]. In addition, EV miR-1290 was found to be a biomarker in ovarian carcinoma [71]. MiR-1290 was not detected or had a very low expression in nEVs, normoxic and hypoxic whole cell lysates (data not shown), which means that this miRNA is specifically loaded into hEVs. We are currently investigating target genes of miR-1290 and transcriptional networks in which this miRNA might be involved. The miRNA contents of EVs and WCLs differed considerably indicating that distinct miRNAs are exported into EVs. Interestingly, upon application of hypoxia, many miRNAs are differentially regulated that are retained inside the cell (Figure 6, WCL) while only few were present in the cell and in EVs (miR-210-3p, miR-92a-1-5p). Overall, cell line-specific expression patterns of miRNAs were apparent, reflecting the high intrinsic heterogeneity of melanoma cells, which were not influenced by presence of either the BRAF or NRAS mutations. 

The migration and invasion assays have shown that A375 hEVs but not nEVs induced invasion of A375 cells and NHDFs compared to A375 nEVs, while 501Mel hEVs increased invasion of NHDFs at early time points only. This could be due to the increased expression of PRMT5 in the melanoma hEVs, as PRMT5 well as RNA have recently been shown to regulate cancer cell invasion [72,73,74]. Here, we did not observe an effect of nEVs or hEVs on proliferation in normoxic conditions, whereas Patton et al. have recently shown that hEVs derived from pancreatic cancer cells can promote proliferation, however under hypoxia [75]. Cells grown under hypoxia or normoxia also did not take up more or less EVs (Figure S10), an observation which we quantified and reproduced several times (data not shown). Taken together, the different EV cargos that are altered under hypoxia did not influence the overall growth behaviour of cancer and surrounding cells. 

In summary, we isolated EVs from melanoma cell lines carrying either the BRAF V600E or an NRAS mutation under normoxia and hypoxia. We generated whole proteome and miRNome data in order to get an overview of differentially expressed proteins and miRNAs under hypoxia in WCLs and EVs (Figure S8). We found a secreted hypoxic signature of specific proteins (AKR7A2, DDX39B, EIF3C, FARSA, PRMT5, VARS) and miRNAs in EVs, which could be further exploited as biomarkers for progression of disease. The proteins present in melanoma hEVs could lead to an increase of glycolysis in recipient cells and transfer mRNA splicing components. Among the miRNAs differentially expressed in melanoma hEVs, miR-1290 is a new and secreted hypoxic miRNA while miR-92a and miR23a/b were found to be downregulated in melanoma hEVs. Some of the proteins (e.g., PRMT5) and miRNAs (miR-210, miR-1290) of the secreted hypoxic signature might represent promising melanoma biomarkers and interesting players involved in shaping the melanoma microenvironment.

## 4. Materials and Methods 

### 4.1. Cell Lines and Cell Culture

A375 melanoma cells were purchased from ATCC, MelJuso and IPC298 cells were purchased from DSMZ (Deutsche Sammlung von Mikroorganismen und Zellkulturen, Braunschweig, Germany) while the 501Mel melanoma cell line was obtained from Ruth Halaban (Dermatology department, Yale School of Medicine, New Haven, CT, USA). Normal Human Dermal Fibroblasts (NHDFs) were obtained from Heike Hermanns (University Hospital Würzburg, Würzburg, Germany). All melanoma cell lines were cultured in RPMI + Glutamax (Lonza BioWhittaker, Basel, Switzerland) + 10% FBS and 1% PS (10,000 U/mL Penicillin and 10,000 U/mL Streptomycin, Lonza BioWhittaker, Basel, Switzerland) while NHDFs were cultured in DMEM + 10% FBS and 1% PS (10,000 U/mL Penicillin and 10,000 U/mL Streptomycin (PS, Lonza BioWhittaker, Basel, Switzerland). All cells were grown at 37 °C in a humidified atmosphere at 5% CO_2_. Cells were regularly tested to be mycoplasma free. Hypoxia treatment was performed at 37°C in a water-saturated atmosphere at 5% CO_2_ in a hypoxia station (Invivo_2_ 400, Ruskinn Technology Ltd, Bridgend, UK) at 1% O_2_.

### 4.2. Small Extracellular Vesicle Isolation

EV donor cells (A375, 501Mel, MelJuso and IPC298) were slowly adapted to serum-free medium (UltraCulture, Lonza BioWhittaker, Basel, Switzerlandlocation). Culture supernatants (100 mL) from cells cultured for 72h under normoxia or hypoxia were harvested, centrifuged for 10 min at 400× *g* followed by centrifugation for 30 min at 2000g to remove cells and cell debris. EVs were isolated by ultracentrifugation (70 min at 110,000g, 4 °C) in an Optima MAX-XP Ultracentrifuge (Beckman Coulter, Brea, CA, USA) using a MLA-55 fixed rotor followed by flotation on an Optiprep cushion (17%, Axis-Shield, Dundee, UK) for 70 min at 100,000g at 4 °C using a swinging MLS-50 rotor. After a PBS wash (110,000g, 70 min), EVs were resuspended in PBS and frozen at -80 °C. EV amounts used in the different experiments were based on protein quantification using the DC protein assay (Biorad, Hercules, CA, USA) according to the manufacturer’s instructions. To label EVs, culture supernatants were processed as described above. After ultracentrifugation at 110,000g, the pellet was resuspended in 250 µL of PBS and stained with 5 µL of PKH67 (Sigma-Aldrich, Saint-Louis, MO, USA) for 30 min at 37 °C. To remove excess dye, the suspension was loaded on an Optiprep cushion, followed by a PBS washing step.

### 4.3. Nanosight Analysis

Size distribution profiles (nm) and concentration measurements (particles/mL) of isolated EV samples were obtained using the NanoSight NS300 instrument (NanoSight Technology, Malvern, UK). All samples were diluted 1000- to 2000-fold in PBS to achieve the ideal particle-per-frame value (20–50 particles/frame). Sample injection into the NanoSight was done with a syringe pump set at 60 µL/s speed at room temperature. The camera (Scmos) was set with a gain of 73 and a shutter of 696 (camera level 10). For each measurement, three 1-min videos were captured, representing 4494 frames, and analysed with the nanoparticle tracking software NTA 3.3 Dev Build 3.3.301 with a detection threshold set at 3. The capture and analysis settings used were manually set according to the manufacturer’s instructions (Nanosight NS300 User manual MAN0541-02-EN).

### 4.4. Western Blot Analysis and Antibodies

Cell lysis was performed at 4 °C using ice cold RIPA lysis buffer containing 25 mM Tris/HCl pH 7.4, 150 mM NaCl, 1% NP40, 0.5% Sodium Deoxycholate, 0.1% SDS. Protein extracts were analysed by SDS-PAGE and Western blotting. ECL signals were detected as described before [76]. The following antibodies were used: α-tubulin (Thermo Fischer Scientific, Waltham, MA, USA), CD63 (Merck Millipore, Burlington, MA, USA), ALDOA, calnexin, FXR2, PKM2, PRMT5 (Cell Signaling, Danvers, MA, USA), CD81 (Biolegend, San Diego, CA, USA), HIF1α (BD Biosciences, Franklin Lakes, NJ, USA), CD9, Tsg101 (Abcam, Cambridge, UK), IPO11 (Novus Biologicals, Centennial, CO, USA).

### 4.5. Mass Spectrometry Based Proteomics

#### 4.5.1. Analysis of the cell proteome

The protocol for analysis of the cell proteome was adapted from a previously published protocol for EV proteome analysis [41]. Cells were grown for 48h under normoxia or hypoxia. Then, 10^6^ cells were lysed with 1% sodium deoxycholate (SDC) in the presence of 10 mM DTT (in 100 mM Tris buffer, pH 8) then sonication cycles on ice were performed. After reduction of disulfide bonds for 1 h at 37 °C, proteins were alkylated with 25 mM iodoacetamide for 1 h at 37 °C, both carried out in the dark. Debris were pelleted by centrifugation for 20 min at 16,000 × g. Protein concentration was assessed by BCA protein assay (Thermo Fisher Scientific, Waltham, MA, USA). Then, 50 μg protein was digested using endopeptidase LysC (1/80, mass spectometry grade, Wako-Fujifilm, Osaka, Japan) followed by Trypsin (1/40, Promega, Madison, WI, USA) overnight, both at 37 °C. Formic acid (FA) was added to a final concentration of 1% to precipitate. SDC pellets were washed three times and combined with the initial supernatant. The peptide supernatants were desalted by solid phase extraction using a C_18_ cartridge (Sep-Pak®, 1cc, Waters Corporation, Milford, MA, USA), eluted with 50% acetonitrile (ACN)/0.1% FA, and dried in a speedvac concentrator (Concentrator plus, Eppendorf, Hamburg, Germany). The dried sample was reconstituted in 100 μL of 1% ACN/0.05% TFA in LC-MS water for LC-MS analysis.

#### 4.5.2. Analysis of the EV proteome 

Similarly to the cell pellets, EV samples equivalent to 50 μg protein were reduced with 10 mM DTT in the presence of 1% SDC (100 mM Tris buffer, pH 8) for 1 h at 37 °C. After reduction, proteins were alkylated with 25 mM iodoacetamide for 1 h at 37 °C, in the dark, until quenched by 10 mM N-acetyl cysteine for 30 min, at room temperature. Protein extraction was performed by methanol/chloroform precipitation. Briefly, 150 μL EV sample were mixed with 1 mL of a methanol:chloroform:water (2:1:2) solution, and vortexed thoroughly, before centrifugation for 5 min at 5000 × g. The upper layer of the solution was removed and 600 μL methanol were added. After vortexing followed by centrifugation for 30 min at 16,000 × g, the supernatant was removed to recover the protein pellet. The air-dried pellet was reconstituted in 1% SDC (100 mM Tris buffer, pH 8.8) for trypsin digestion for 16 h, at 37° C. SDC precipitation and desalting were carried out as described above. 

#### 4.5.3. LC-MS/MS analysis

Peptide concentration of the reconstituted sample was quantified measuring the absorbance at 205 nm using Nanodrop^TM^ One. The equivalent of 200 ng peptide sample was injected for analysis using an Ultimate 3000 RSLCnano reverse-phase liquid chromatography (LC) system (Thermo Fisher Scientific, Waltham, MA, USA). Peptides were first trapped for pre-concentration (PepMap, 2 cm × 75 μm ID, C18, 3 μm, 100 Å) and subsequently separated (PepMap, 15 cm × 75 μm ID, C18, 2 μm, 100 Å) in column switching configuration. A linear gradient of 2–35% solvent B at 300 nL/min was applied over 66 min for peptide elution (solvent A: 100% LC-MS water/0.1% FA, solvent B: 100% ACN/0.1% FA). Analysis was carried out using a Q-Exactive Plus mass spectrometer equipped with a nano-electrospray source (Thermo Fisher Scientific, Waltham, MA, USA). For MS/MS analysis, data-dependent acquisition (DDA) was performed fragmenting the 12 most intense peptide ions with a dynamic exclusion time of 20 s. MS1 spectra were generated with a resolving power of 60,000 and 15,000 for MS2. Target automatic gain control (AGC) for MS2 was set to 1e5, and the fill time was 45ms maximum. 

#### 4.5.4. Protein identification

For protein identification of LC-MS/MS files, a protein database search was carried out using Andromeda search engine within MaxQuant (Version 1.6.7.0, https://www.maxquant.org/) with 1% FDR for peptides and proteins. Carbamidomethylcysteine was set as fixed modification. Methionine oxidation and protein N-terminal acetylation were set as variable modifications. Canonical human proteome database UP000005640 was used for database search (downloaded 26.02.2019, https://www.ebi.ac.uk/reference_proteomes/). A maximum of two missed cleavages were allowed and label-free quantification (LFQ,) and match between runs (MBR) was performed with default settings.

### 4.6. miRNA Profiling by qPCR Arrays

RNA of EVs corresponding to 50 µg protein was extracted using the microRNA purification kit (Norgen Biotek, Thorold, ON, Canada) according to the manufacturer’s instructions with the use of three spike-in controls, cel39, cel238, cel 54 (Qiagen, Hilden, Germany) in order to assess RNA extraction. Then, 5 µL EV RNA (out of 20 µL total eluted volume) was reversed-transcribed using the HiSpec buffer with the miScriptII RT kit (Qiagen, Hilden, Germany). Due to the generally low RNA amounts extracted from EVs, a whole miRNome pre-amplification step was necessary before performing the qPCR arrays. The miRNA profiling with the human miRnome miScript miRNA qPCR arrays (v.16, 1066 miRNAsQiagen, Hilden, Germany) and subsequent data analysis were performed according to the manufacturer’s instructions and as described in [77]. Raw data files of all qPCR arrays are available upon request. To validate qPCR array data, EV RNA was re-extracted in three biological replicates, pre-amplified and reversed-transcribed as described above. qPCR was carried out using specific 10× miScript primer assays (Qiagen, Hilden, Germany) for individual miRNAs on a CFX96 Detection System (Biorad, Hercules, CA, USA). The spike-in controls and the target miRNAs were analysed in parallel for each sample. All samples were assayed in triplicate.

### 4.7. miRNA Profiling by miRNA Microarrays

For microarray analyses, total RNA was extracted in triplicate using the Quick-RNA™ Mini-Prep Kit (ZYMO Research, Irvine, CA, USA) following the manufacturer’s instructions. RNA purity and quality were assessed using the NanoDrop2000 Spectrophotometer (Thermo Fisher Scientific, Waltham, MA, USA). The Affymetrix miRNA v4.1 platform was used. Raw microarray data are accessible in the ArrayExpress database (https://www.ebi.ac.uk/arrayexpress/) under the accession number E-MTAB-8577. Data analysis is described below (see bioinformatics analysis, Section 4.13).

### 4.8. miRNA Mimic Transfections and Detection of Target Expression by qPCR and WB

First, 5 × 10^4^ and 2.5 × 10^4^ cells/well were seeded in 12- and 24-well culture plates (Greiner Bio-One, Kermsmünster, Austria) in technical triplicates, respectively. Then, 24h post-seeding, cells were transfected with 25nM miR-23a*, miR-23b* or miR-1290 miRCURY LNA miRNA mimics or negative control mimic (Qiagen, Hilden, Germany) using the HiPerfect transfection reagent as described before [78]. RNA and protein lysates were harvested at 24, 48 and 72h after transfection. Total RNA was extracted using the Quick-RNA™ Mini-Prep Kit (ZYMO Research, Irvine, CA, USA) following the manufacturer’s instructions with on-column DNase treatment. RNA purity and quality were assessed using the NanoDrop2000 Spectrophotometer (Thermo Fisher Scientific, Waltham, MA, USA). Reverse transcription was performed from 100–250ng RNA in 10μL reaction volume using the miScript II RT kit (Qiagen, Hilden, Germany) according to protocol. qPCR was carried out on a CFX384 Detection System (Biorad, location) using specific 10× miScript primer assays (Qiagen, Hilden, Germany) for individual miRNAs to control for successful mimic transfection and RT2 qPCR Primer Assays (Qiagen, Hilden, Germany) for IPO11 and FXR2 mRNAs. Specificity of the qPCR primers was assessed by a post-qPCR melting curve analysis. Cq-values for mRNA and miRNA species were normalised to three reference genes: TBP, HPRT and PPIA for mRNAs and RNU1A, RNU5A and SCARNA17 for mature miRNAs using geNorm. Protein lysates were analysed as described in Section 4.4.

### 4.9. Confocal Microscopy

A375 or 501Mel cells were grown on glass coverslips for 24 h. Then, 5 µg of PKH67-labelled EVs were added to the cells for 16 h. Cells were washed with PBS and fixed with 4% paraformaldehyde in PBS for 30 min at room temperature. The coverslips were washed three times in PBS-0.5% Triton X-100. Next, cells were permeabilised with PBS-0.5% Triton X-100 for 10 min at room temperature, and blocked in PBS plus 5% milk for 30 min. Coverslips were washed three times with PBS-0.5% Triton X-100 and treated with anti-phalloidin-Alexa 546 (Sigma-Aldrich, Saint-Louis, MO, USA) to stain the actin filaments and DAPI (Invitrogen, Carlsbad, CA, USA) to stain the nuclei for 1 h at room temperature. Coverslips were washed and mounted with Mowiol (Sigma-Aldrich, Saint-Louis, MO, USA). The cells were visualised by Andor Revolution Spinning Disk confocal microscopy, mounted on a Nikon Ti microscope (60× oil objective), and the images were analysed with ImageJ software.

### 4.10. EV Uptake Experiments

For EV uptake experiments, A375 or 501Mel cells were grown for 24 h on 96-well Matriplates pre-treated with Poly-L-lysine hydrobromide (Sigma-Aldrich, Saint-Louis, MO, USA). Then, 5µg of PKH67-labelled EVs were added to the cells for 16 h. Cells were washed with PBS and fixed with 4% paraformaldehyde in PBS for 30 min at room temperature and were stained as described above. Cells were visualised by Cytation 5 (BioTek instruments, Winooski, VT, USA). The images were analysed with the Gen5 Image Prime software (BioTek instruments, Winooski, VT, USA) and the Gen5 spot counting module (BioTek instruments, Winooski, VT, USA).

### 4.11. Invasion and Migration Assays

A375, 501Mel cells and NHDFs were seeded at a density of 3 × 10^4^, 3.5 × 10^4^ and 16.7 × 10^4^ cells/well, respectively, in collagen I-coated (200 µg/mL, Merck Millipore, Burlington, MA, USA) 96-well plates (Essen Imagelock, Essen Bioscience, Ann Arbor, MI, USA). Then, 24 h after seeding, when cells had reached 80–90% confluence, a wound was scratched across each well with the Cellplayer 96-well woundmaker (Essen Bioscience, Ann Arbor, MI, USA). After the scratching, 5 µg nEVs or hEVs were added per well. Migration and invasion were studied as previously described in [78].

### 4.12. Proliferation Assays

A375, 501Mel melanoma cells and NHDFs were seeded in 96-well plates in complete medium. The melanoma nEVs or hEVs were added at different concentrations (22.5, 45 and 90 µg/mL) to the cells. A blank control (medium only), as well as an untreated control for each cell line, were included. After 48h, cell viability was assessed using PrestoBlue^®^ cell viability reagent (Thermo Fisher Scientific, Waltham, MA, USA) and fluorescence was measured using the microplate reader CLARIOstar (BMG-LABTECH, Ortenberg, Germany). Growth was expressed as percentage of living cells upon EV treatment as compared to the untreated control (relative cell viability (%)). The experiment was performed in biological triplicates, with three technical replicates for each biological replicate.

### 4.13. Bioinformatic Analysis

#### 4.13.1. Differential Expression Analysis of Proteins in Small Extracellular Vesicles

Data were processed using Maxquant (version 1.6.7.0, https://www.maxquant.org/) and LFQ.intensities were imported into R (ver. 3.6.0, https://www.r-project.org/, [79]) for further analysis using DEP library and workflow [80]. Proteins with indicators Reverse “+” and Potential.contaminant “+”, and samples that appeared as outliers in the PCA, namely “EV3HMelJuso”, “EV3NMelJuso”, “WCL1NMelJuso”, “WCL1HMelJuso” were filtered out. Due to notable differences in the amounts of identified proteins, WCL and EV data were analysed separately. For each compartment, the following process was applied: (1) data were normalised using vsn [81], (2) proteins not present in more than two samples were removed and (3) missing values were imputed using a mixed imputation: MinDet type for non-random ones, knn for random ones, (4) differential expression between hypoxia and normoxia was estimated in each cell line using linear modelling and eBayes procedure. Proteins with adjusted *p*-values lower than 0.05, and fold change larger than 2 (or smaller than 1/2) were considered significant. From that selection, only the proteins with less than two imputed values in the compartment were analysed further.

#### 4.13.2. miRNA Microarray Analysis

RMA-preprocessed data were obtained from Aros Applied Biotechnology A/S and were further analysed in R and Bioconductor (ver. 3.9, [79]). Packages tidyverse [82] and ggplot2 [83] were used for data grooming and representation. Affymetrix controls and miRNAs with less than three expressed values (<2) were filtered out before analysis. This resulted in a 699 probe set matrix. Differential expression under hypoxia in the different cell lines was obtained using limma [84]. MiRNAs with adjusted *p* < 0.05 and fold change > 1.2 (or lower than 1/1.2) were selected as significant. 

#### 4.13.3. qPCR miRNA Selection

For miRNA expression in EVs, qPCR data were normalised per plate to the mean of expressed miRNAs in that sample [77]. A grand mean was added to keep the same scale. Fold change was computed as the ratio hypoxia/normoxia. 

#### 4.13.4. miRNA Target Analysis

Validated targets were obtained using SpidermiR [85], which contains a recent version of miRTarBase (v7, 2017, [86]). Predicted targets were retrieved from miRNAtap [87], which aggregates and ranks results from Targetscan (v7.1, [88]). For predicted targets, only the top 100 results for each miRNA were kept. 

#### 4.13.5. Functional Analysis

All analyses were performed in R environment [79]. Functional enrichment analyses of proteins (corresponding genes) and graphing were performed using clusterProfiler library [89]. Curated gene sets from mSigDB, version 7.0 were used as gene set source (https://www.gsea-msigdb.org/gsea/msigdb/index.jsp). In particular, hallmark gene sets [15] were used, and GO biological processes, restricting to processes with a size between 50 and 500. For top and differentially expressed proteins, significant results were selected on the basis of a false discovery rate (FDR)-corrected *p*-value cut-off of 0.05 for hallmarks, 0.005 for GO BP. 

#### 4.13.6. Kaplan–Meier Curves

The correlation between specific gene expression and patient survival was analysed using the platform survexpress (http://bioinformatica.mty.itesm.mx:8080/Biomatec/SurvivaX.jsp) [90]. The dataset of melanoma patients used was SKCM-TCGA Skin Cutaneous Melanoma July 2016 (366 samples).

#### 4.13.7. Analysis of Patient Data 

All data were analysed in R (R version 3.6.1 (5 July 2019), [79]). We used data from the SKCM dataset of TCGA. Expression data (FPKM_UQ units) and clinical data were retrieved using TCGAbiolinks (version 2.14.0, [91]). Expression data were log-transformed to match normality assumptions. Survival analyses were performed using survival (version 3.1.8), [92]). Graphs were plotted using ggplot2 [83]. Before analysis, the following cleaning/filtering steps were applied: (1) only patients with primary diagnosis either “Malignant melanoma, NOS”, “Nodular melanoma” or “Superficial spreading melanoma” were kept in order to retain only the main types of melanoma. (2) Patients with Stage “i/ii nos” or “not reported” were filtered out, and (3) Stage groups were defined as follows: “Early” (Stages 0 and 1), “Intermediate” (Stage 2) and “Late” (Stages 3 and 4). Association between clinical variables and gene expression was estimated by a linear model (ANOVA). Survival was modelled separately for each gene using a Cox regression, including Stage group in the model. All *p*-values were FDR corrected taking into account the number of genes tested (60).

#### 4.13.8. Statistical analysis

Statistical analysis was performed with the GraphPad Prism software (version 8.2.1, GraphPad Software, https://www.graphpad.com/). All data shown are a representative experiment of three biological replicates. Data are shown for a representative experiment ± s.d. and were analysed either with paired Student’s *t*-test or two-way ANOVA coupled with Tukey’s multiple comparison tests.

## 5. Conclusions

In order to get insights into the molecular intracellular content of melanoma cells as well as their secretome under different oxygen concentrations, we profiled miRNomes and proteomes of WCLs and EVs derived from cells grown under normoxia or hypoxia. We found specific intra- and extracellular signatures of differentially expressed miRNAs and proteins responding to hypoxic conditions in four melanoma cell lines, several of which are likely involved in shaping the melanoma microenvironment. Such differentially regulated miRNAs and proteins in EVs from hypoxic cancer cells are currently being further tested as promising biomarkers of disease progression. 

## Supplementary Materials

The following are available online at https://www.mdpi.com/2072-6694/12/3/692/s1. **Figure S1**. Expression of HIF1α in melanoma cells exposed to normoxia (N) or hypoxia (H), **Figure S2**. Number of proteins identified by mass spectrometry in WCL samples or EV samples of four melanoma cell lines (A375, 501Mel, MelJuso and IPC298), **Figure S3**. Graph depicting the number of proteins in EV samples, matching the top 100 proteins commonly identified in EVs (Exocarta), **Figure S4.** (A) Heatmap showing all the identified proteins in hEVs and nEVs or hypoxic and normoxic WCLs of the four melanoma cell lines, without hierarchical clustering and (B) heatmap showing foldchanges of all differentially expressed proteins upon hypoxia, **Figure S5**. (A) Heatmap showing all the identified proteins in hEVs and nEVs or hypoxic and normoxic WCLs of the four melanoma cell lines, without hierarchical clustering and (B) heatmap showing fold changes of all differentially expressed proteins upon hypoxia, **Figure S6**. (A) Graph representing the Gene Ontology “Biological Processes” enriched among the 50 top expressed proteins in nEVs, hEVs, normoxic or hypoxic WCLs, the top five significant categories in each condition are shown. (B) Graph representing the Gene Ontology “Biological Processes” enriched among only the differentially expressed proteins in nEVs, hEVs, normoxic or hypoxic WCLs, **Figure S7**. Expression of hypoxia signature proteins in WCL and EVs of four melanoma cell lines (A375, MelJuso, IPC298, 501Mel), **Figure S8**. Heatmap showing the differentially expressed proteins in hEVs, which are targets of miR-1290 (FXR2) or of miR-23a-5p/miR-23b-5p (IPO11), **Figure S9**. Expression analysis of mRNA and protein levels of FXR2 and IPO11 in melanoma cells transfected with either miR-1290, miR-23a-5p (miR-23a*) or miR-23b-5p (miR-23b*) mimics, **Figure S10**. EV uptake assay, **Table S1**. Differentially expressed genes upregulated in hEVs in at least two melanoma cell lines, which were imputed less than three times, **Table S2**. qPCR validation of selected miRNAs, upregulated (miR-210, miR-1290, miR-323a-5p) or downregulated (let-7d-3p, miR-23b-5p, miR-708-5p, miR-23a-5p) for the four melanoma cell lines (A375, 501Mel, MelJuso and IPC298); **Spreadsheet S1.** Differential expression of proteins identified in by mass spectrometry in WCL samples or EV samples of four melanoma cell lines (A375, 501Mel, MelJuso and IPC298).
