# Supplementary material for: Hypoxia-Induced Adaptations of miRNomes and Proteomes in Melanoma Cells and Their Secreted Extracellular Vesicles
Source: Cancers (Basel). 2020 Mar 14;12(3):692. doi: 10.3390/cancers12030692 (PMC7140034; doi:10.3390/cancers12030692)
Supplement: Supplementary file 1 [file cancers-12-00692-s001.pdf]

Figure S1

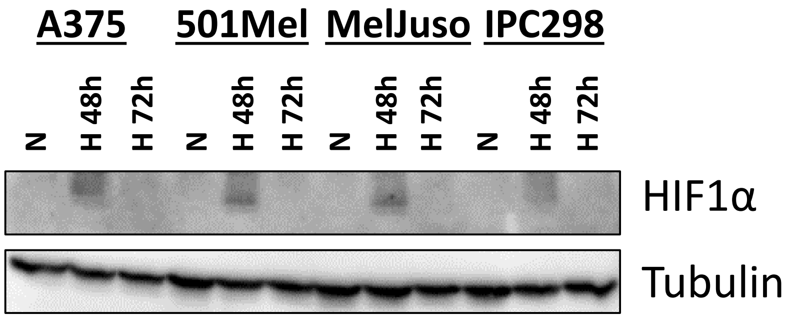

Figure S1. Expression analysis of HIF1α in 4 melanoma cell lines exposed to normoxia (N) or hypoxia (H) (1% O_2 for 48h or 72h) by Western blot.

Figure S2

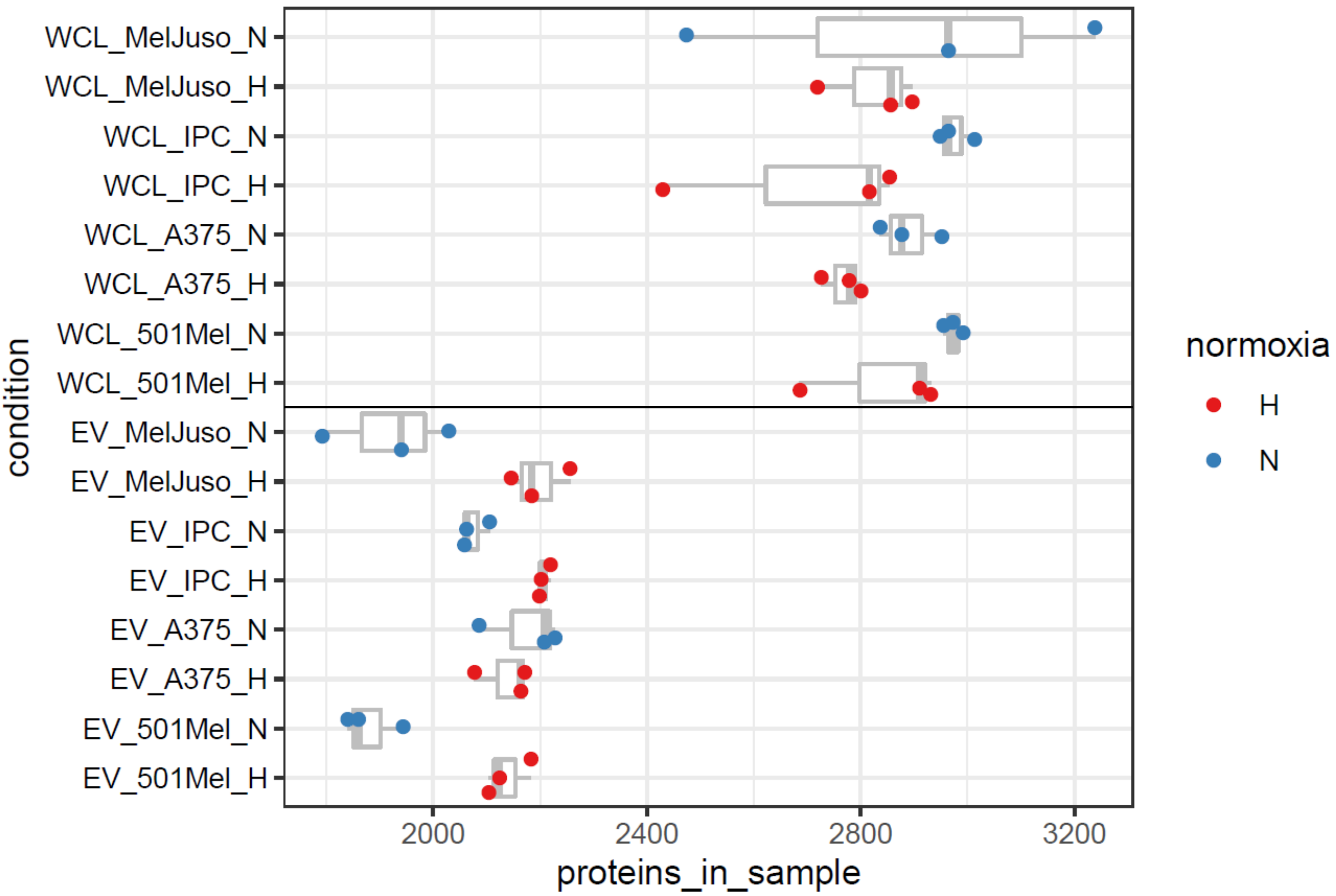

Figure S2. Number of proteins identified by mass spectrometry in WCL samples or EV samples of four melanoma cell lines (A375, 501Mel, MelJuso and IPC298). N: normoxia, H: hypoxia.

Figure S3

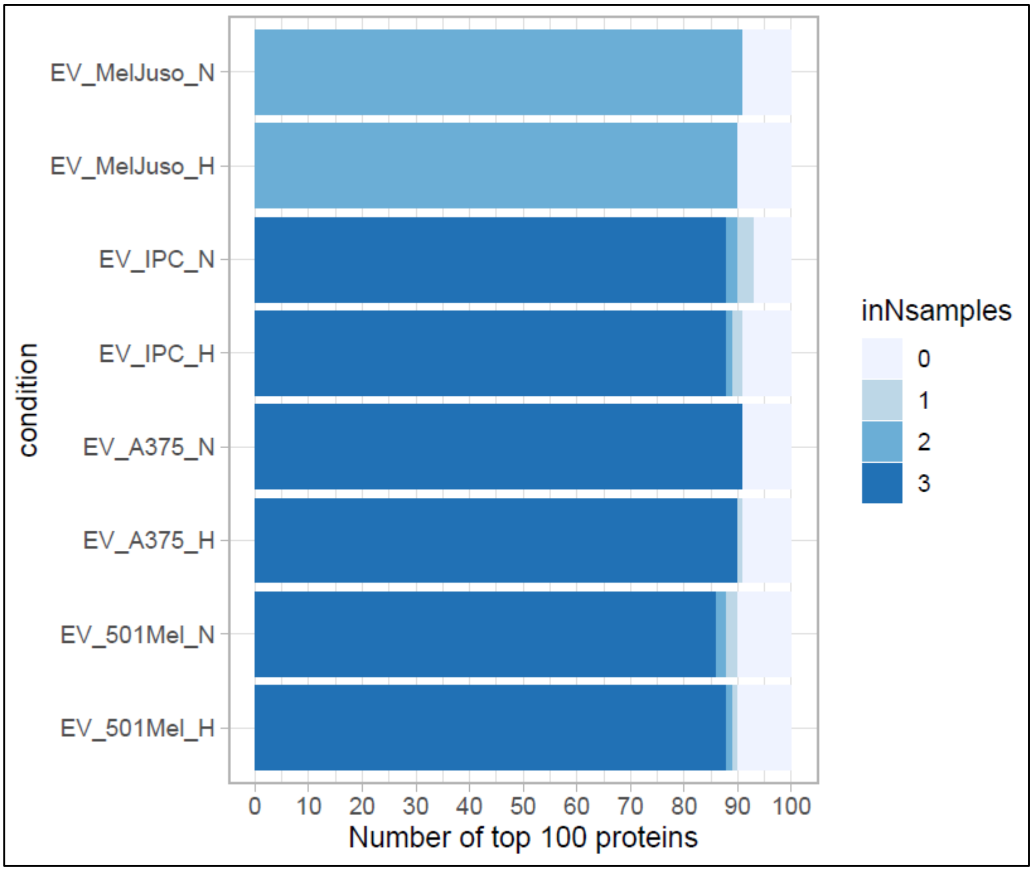

Figure S3. Graph depicting the number of proteins in EV samples, matching the top 100 proteins commonly identified in exosomes (Exocarta). Three biological replicates per melanoma cell line except for MelJuso (2 biological replicates).

Figure S4

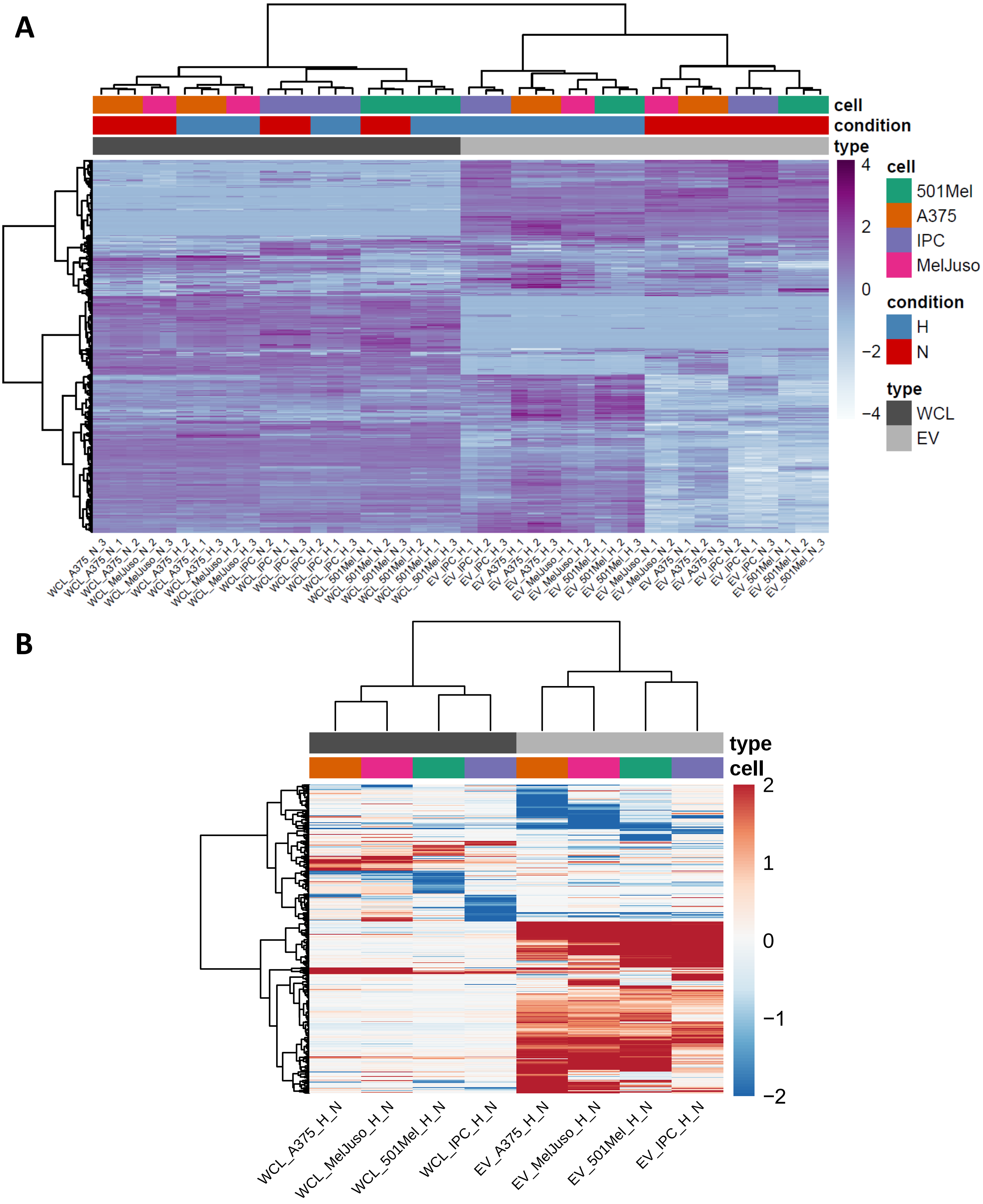

Figure S4. (A) Heatmap showing all the identified proteins in hEVs and nEVs or hypoxic and normoxic WCLs of the 4 melanoma cell lines, without hierarchical clustering and (B) heatmap showing fold changes of all differentially expressed proteins upon hypoxia. Color indicates fold change.

Figure S5

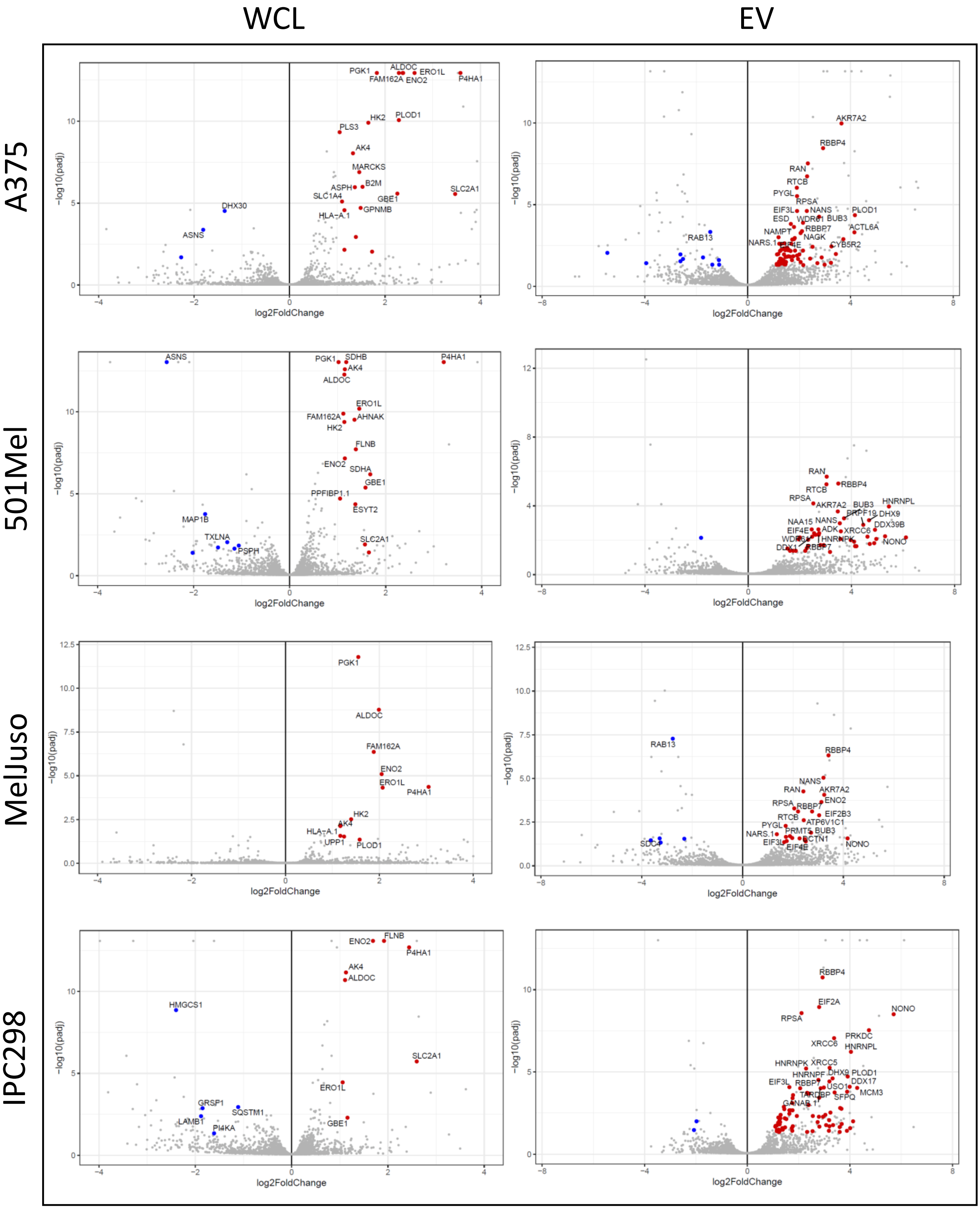

Figure S5. Volcano plots showing the differentially expressed proteins in hEVs and nEVs or hypoxic and normoxic WCLs of the 4 melanoma cell lines.

Figure S6

A

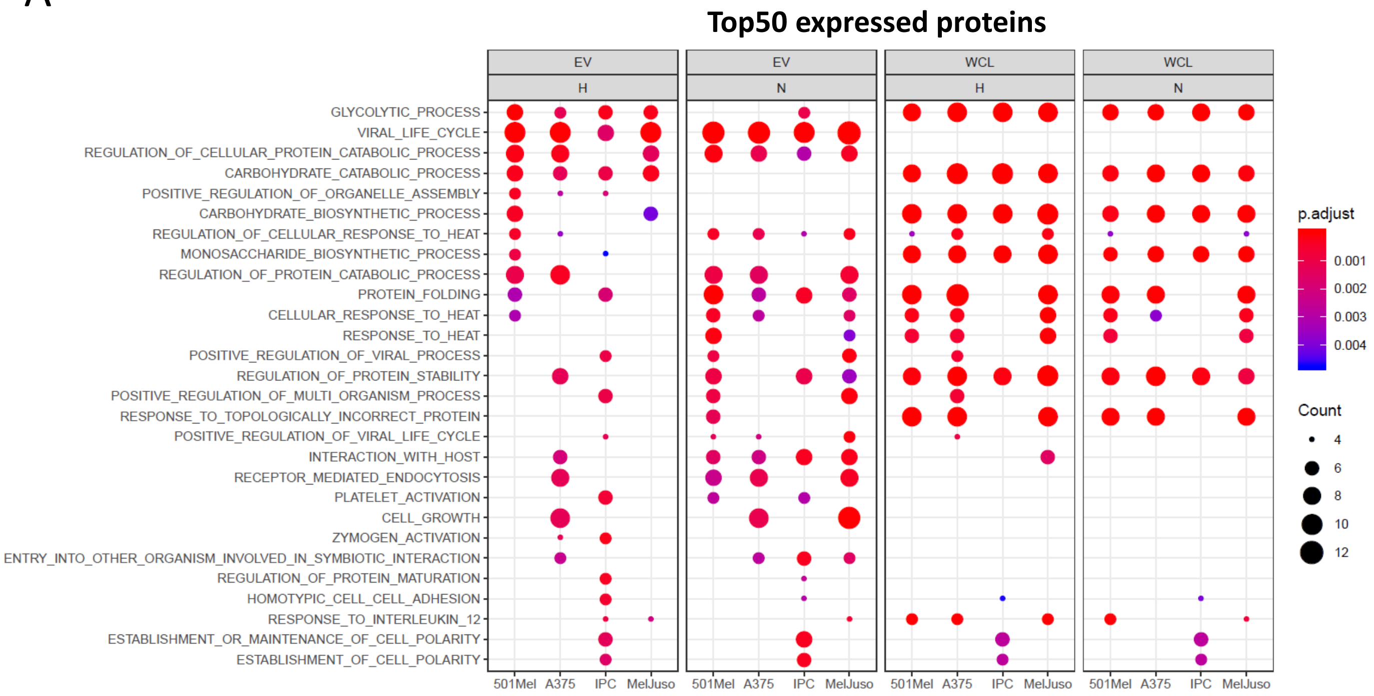

B

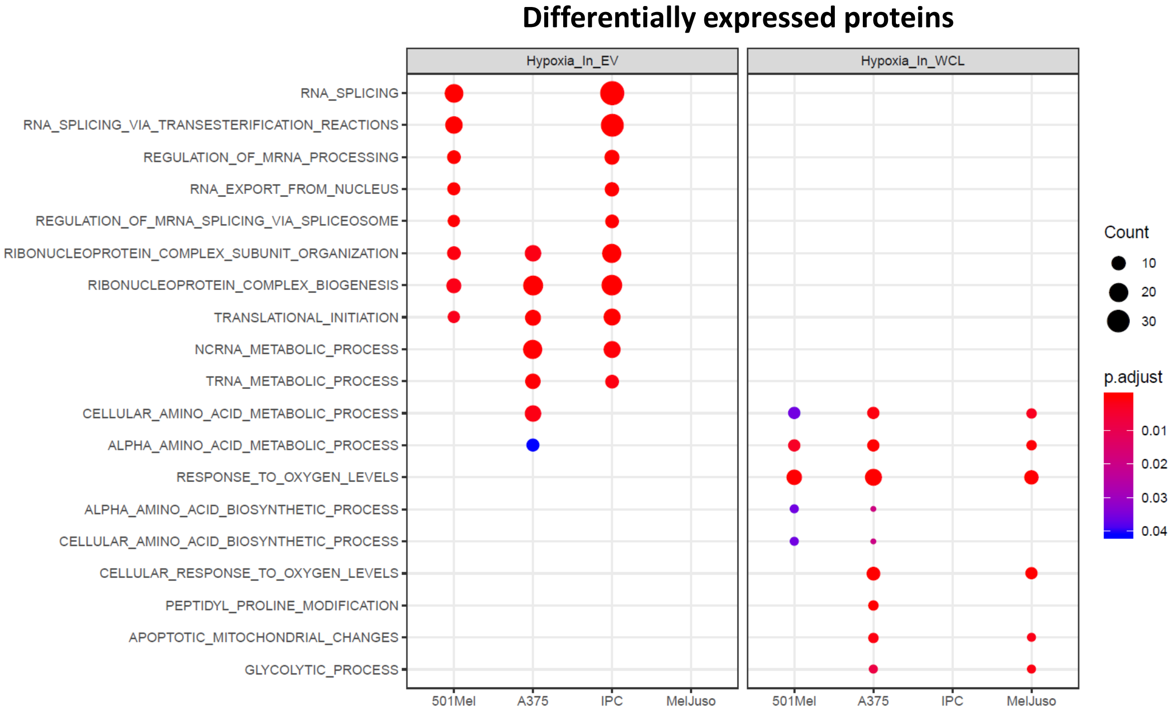

Figure S6. (A) Graph representing the Gene Ontology "Biological Processes" enriched among the 50 top expressed proteins in nEVs, hEVs, normoxic or hypoxic WCLs, the top 5 significant categories in each condition are shown. (B) Graph representing the Gene Ontology "Biological Processes" enriched among only the differentially expressed proteins in nEVs, hEVs, normoxic or hypoxic WCLs, the top 5 significant categories in each condition are shown. Count is the number of genes identified per GO biological process for the indicated compartment. Color shows the adjusted p-value.

Figure S7

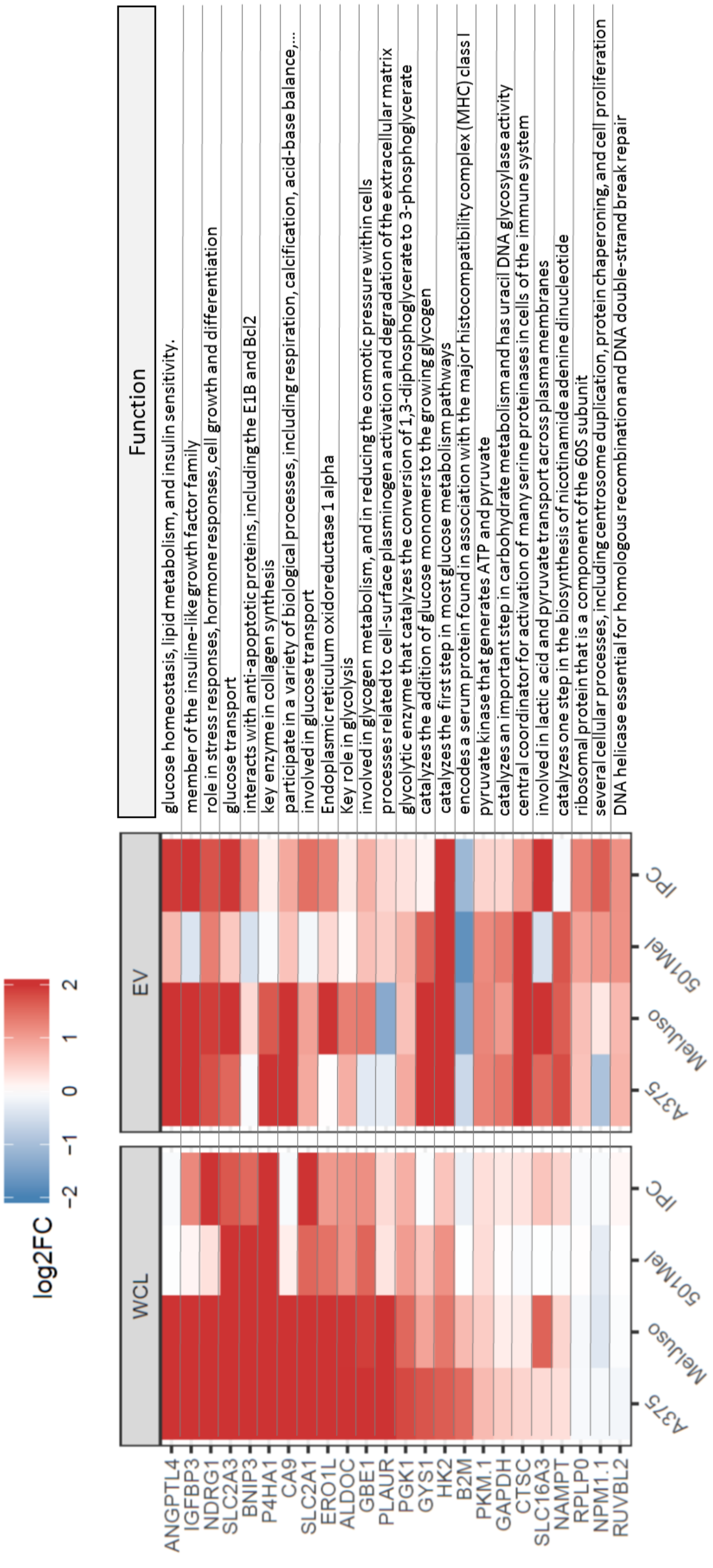

Figure S7. Expression of hypoxia signature proteins in WCL and exosomes of 4 melanoma cell lines (A375, MelJuso, IPC298, 501Mel). Colors show the foldchange in expression relative to normoxia (log2 scale). Proteins are arbitrarily sorted by their expression level in 501Mel WCLs. The function of each gene is summarized on a table adjoining the heatmap (https://www.ncbi.nlm.nih.gov/gene/).

Figure S8

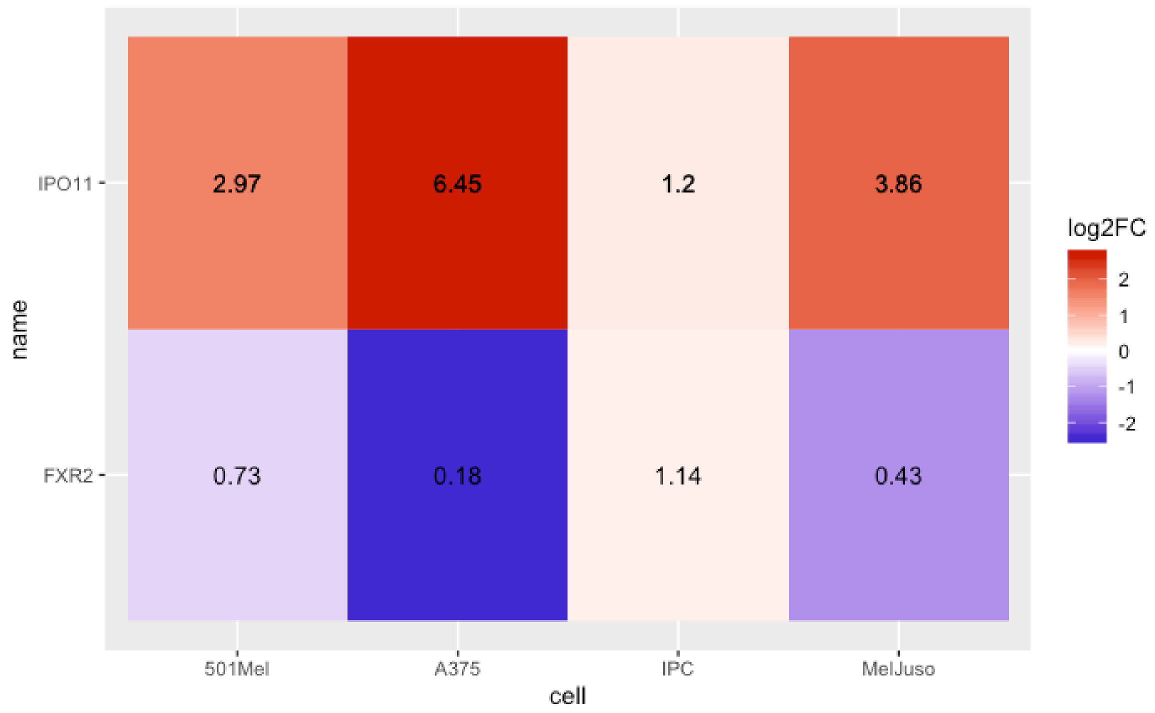

Figure S8. Heatmap showing the differentially expressed proteins (based on MS data) in hEVs compared to nEVs, which are predicted targets of miR-1290 (FXR2) or of miR-23a-5p/miR-23b-5p (IPO11). Numbers indicates the changes in expression relative to normoxia and colors show the foldchange in expression relative to normoxia (log2 scale).

Figure S9

A

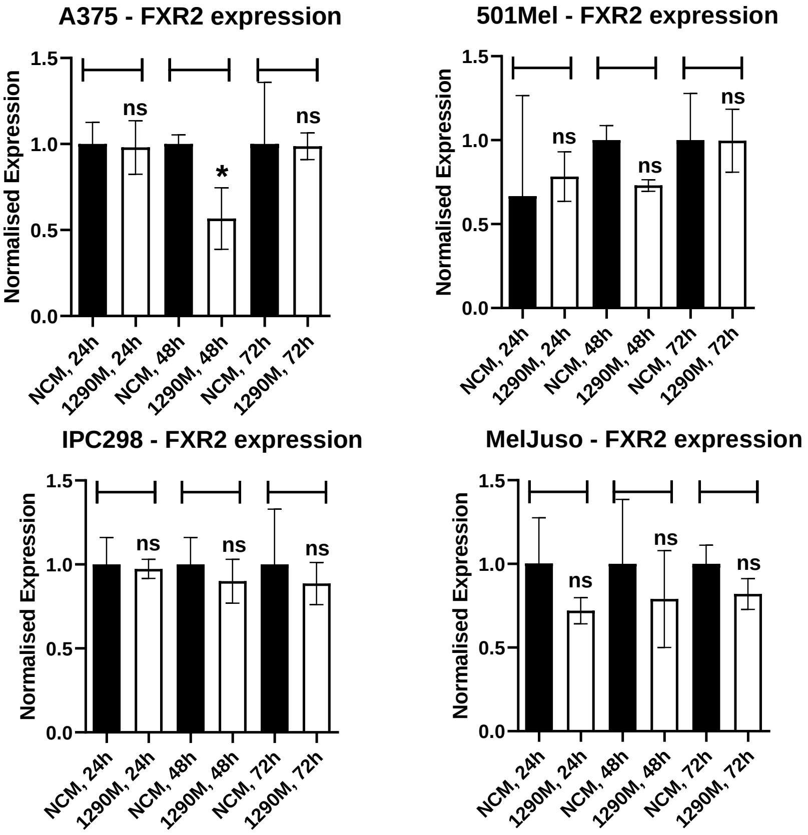
A375 - FXR2 expression
501Mel - FXR2 expression
IPC298 - FXR2 expression
MelJuso - FXR2 expression
Normalised Expression
1.5
1.0
0.5
0.0
ns
*
NCM, 24h
1290M, 24h
NCM, 48h
1290M, 48h
NCM, 72h
1290M, 72h

B

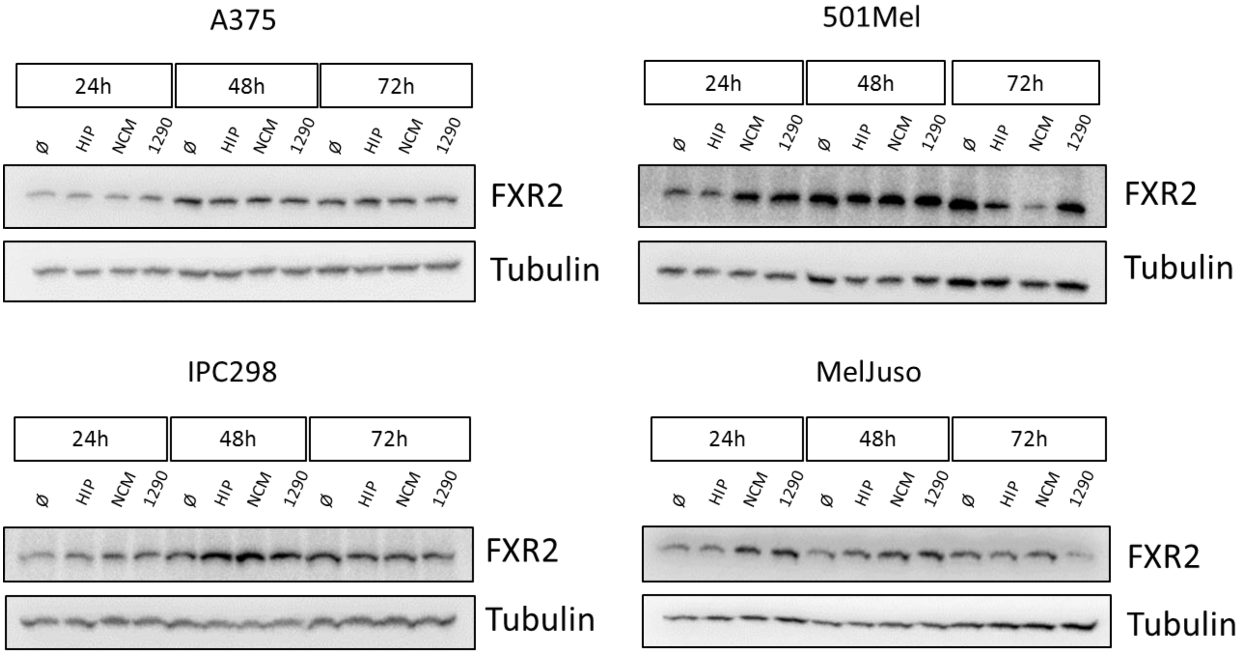
A375
501Mel
IPC298
MelJuso
24h
48h
72h
Ø
HIP
NCM
1290
FXR2
Tubulin

Figure S9

C

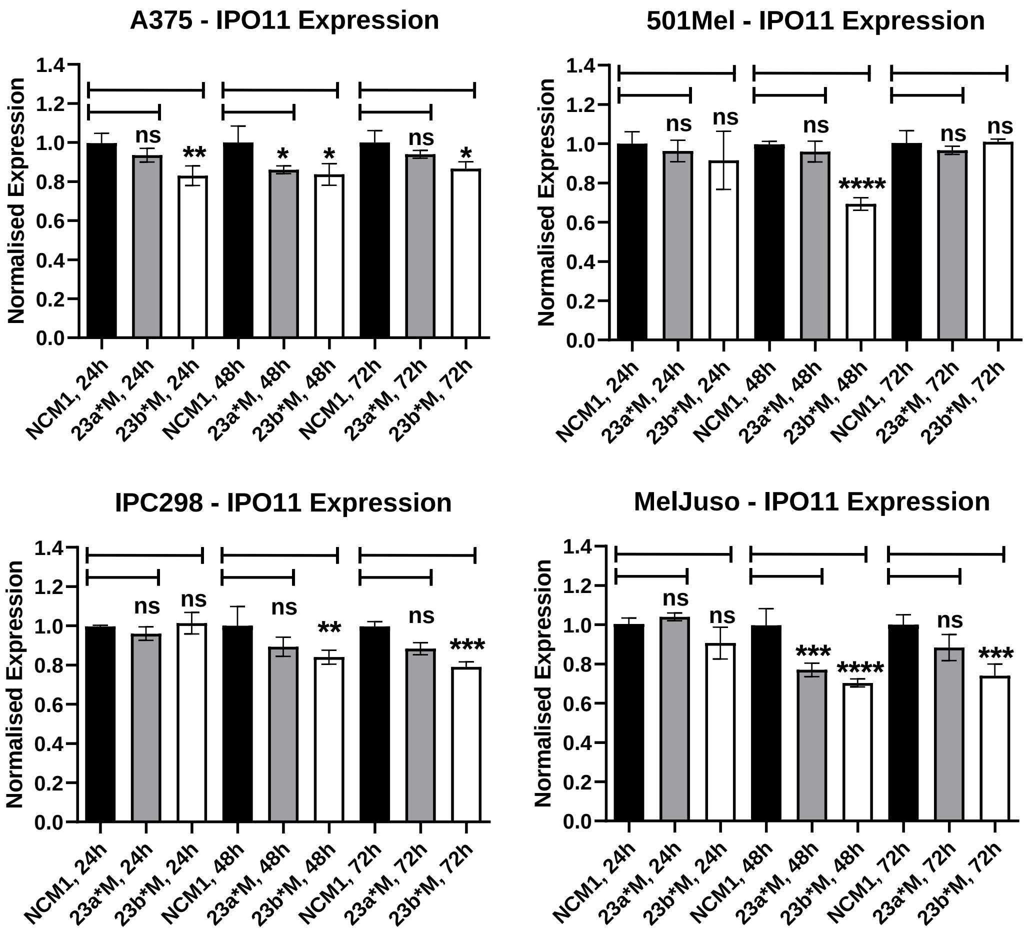
A375 - IPO11 Expression
501Mel - IPO11 Expression
IPC298 - IPO11 Expression
MelJuso - IPO11 Expression
Normalised Expression
ns
**
*
****
***
NCM1, 24h
23a*M, 24h
23b*M, 24h
NCM1, 48h
23a*M, 48h
23b*M, 48h
NCM1, 72h
23a*M, 72h
23b*M, 72h

D

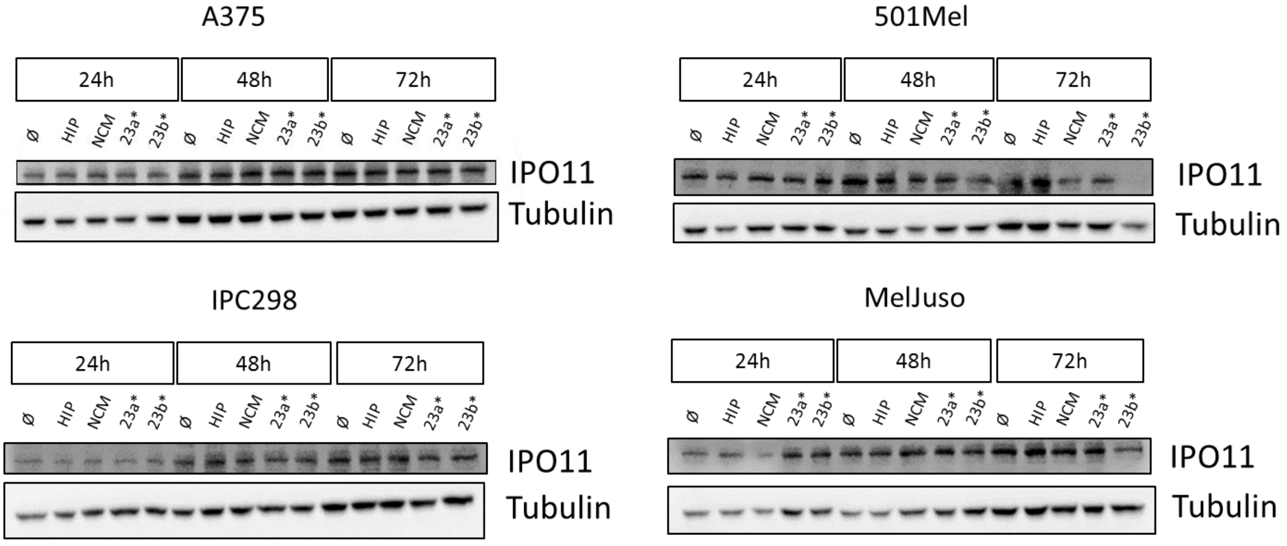
A375
501Mel
IPC298
MelJuso
24h
48h
72h
Ø
HIP
NCM
23a*
23b*
IPO11
Tubulin

Figure S9

Figure S9. Expression analysis of mRNA and protein levels of FXR2 and IPO11 in melanoma cells transfected with either miR-1290, miR-23a-5p (miR-23a*) or miR-23b-5p (miR-23b*) mimics. (A) Normalized expression levels of FXR2 mRNA in 4 melanoma cell lines transfected with miR-1290 mimic or negative control mimic (NCM) at 24, 48 or 72h after transfection. (B) Expression analysis of FXR2 protein level in non-transfected cells (Ø), cells transfected with HiPerfect transfection reagent (HIP) only or cells transfected with miR-1290 mimic or negative control mimic (NCM) at 24, 48 or 72h after transfection by Western blot. (C) Normalized expression levels of IPO11 mRNA in 4 melanoma cell lines transfected with either miR-23a* or miR-23b* mimic or negative control mimic (NCM) at 24, 48 or 72h after transfection. (D) Expression analysis of IPO11 protein levels in non-transfected cells (Ø), cells transfected with HiPerfect transfection reagent (HIP) only or transfected with either miR-23a* or miR-23b* mimic or negative control mimic (NCM) at 24, 48 or 72h after transfection by Western blot. *: $p < 0.05$, **: $p < 0.01$, ***: $p < 0.001$, compared to NCM treatment for each time-point. Statistical analysis was performed using one-way ANOVA coupled with Dunnett’s multiple comparison test.

Figure S10

A

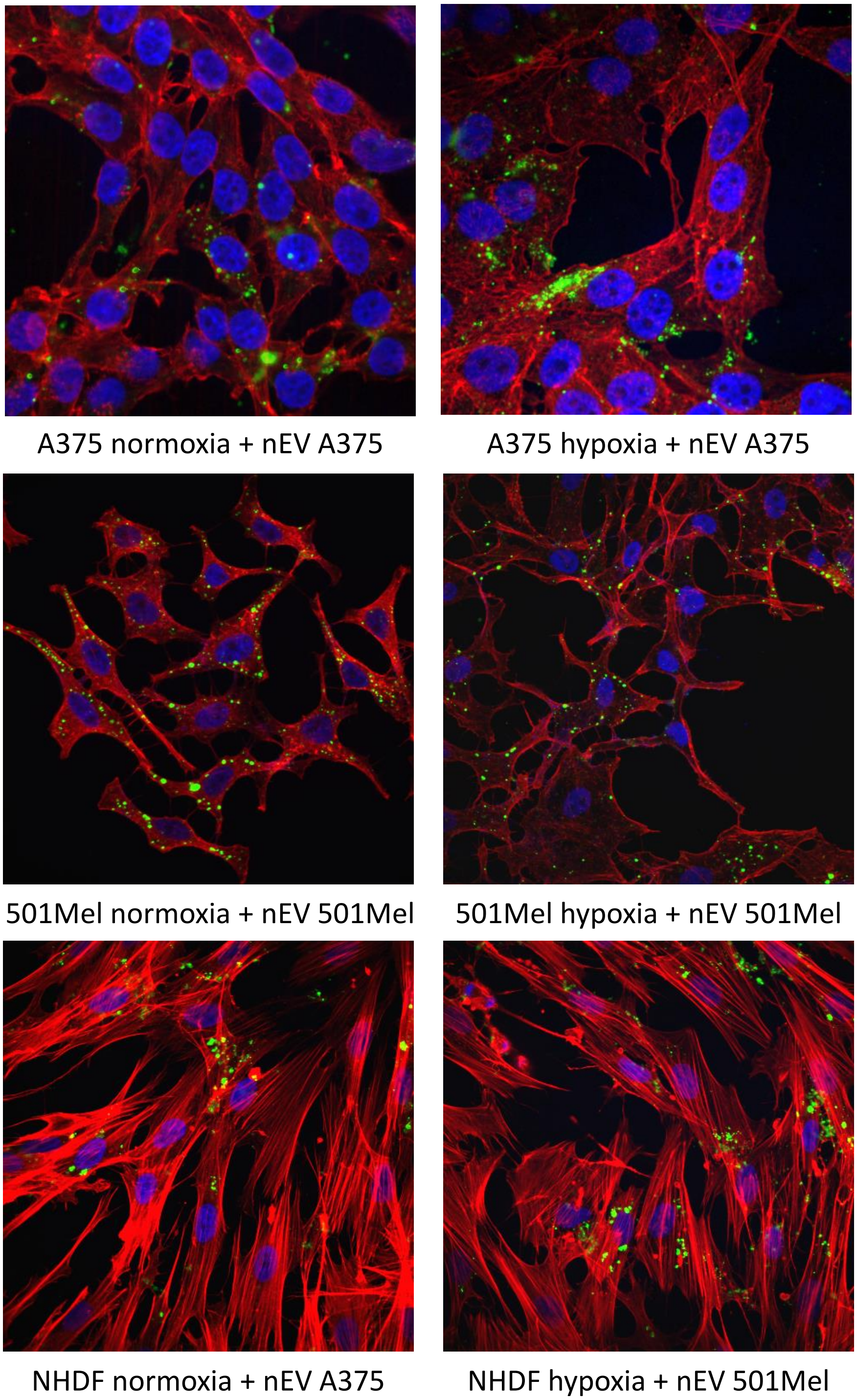
A375 normoxia + nEV A375
A375 hypoxia + nEV A375
501Mel normoxia + nEV 501Mel
501Mel hypoxia + nEV 501Mel
NHDF normoxia + nEV A375
NHDF hypoxia + nEV 501Mel

Figure S10

B

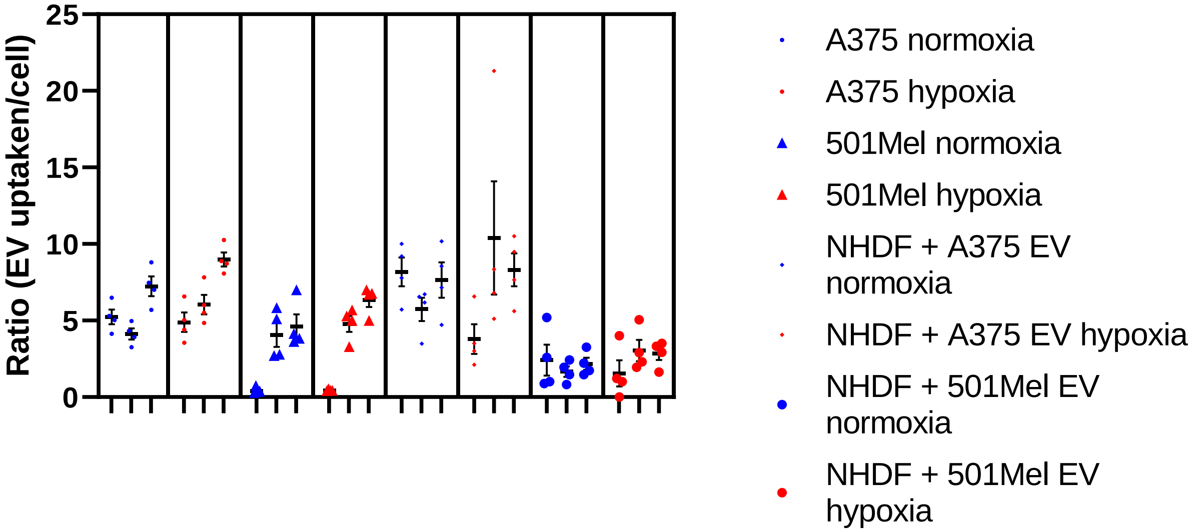

Figure S10. EV uptake assay. (A) Confocal microscopy of A375, 501Mel and NHDF cells, under normoxia or hypoxia, after 16h incubation with A375 and/or 501Mel nEV labelled with PKH67. (B) Ratio of EVs uptaken/cell for all co-culture experiments as described in (A). Spots were counted using an automatic counting software on Cytation5 pictures.

Table S1

Table S1. Differentially expressed proteins in hEVs in at least 2 melanoma cell lines, which were imputed less than three times. Log rank p values for survival risk difference between high and low expression of the indicated gene. Significant differences for patient survival for the proteins upregulated in hEVs are p-values ≤0.055 which are marked in red.

Protein	p-value	adjusted.p	Protein name
ACTL6A	0.6858	0.8992	Actin-like protein 6A
ADK	0.2598	0.6335	Adenosine kinase
**AKR7A2**	0.0421	0.3380	Aflatoxin B1 aldehyde reductase member 2
ARCN1	0.1366	0.5373	Coatomer subunit delta
AURKB	0.0630	0.3380	Aurora kinase B
BUB3	0.3948	0.6755	Mitotic checkpoint protein BUB3
CBR1	0.8194	0.9114	Carbonyl reductase [NADPH] 1
COPA	0.9205	0.9698	Coatomer subunit alpha;Xenin;Proxenin
COPB1	0.0012	0.0765	Coatomer subunit beta
COPB2	0.1199	0.5053	Coatomer subunit beta
DDX1	0.1685	0.5408	ATP-dependent RNA helicase DDX1
DDX17	0.1808	0.5408	Probable ATP-dependent RNA helicase DDX17
**DDX39B**	0.0554	0.3380	Spliceosome RNA helicase DDX39B
DHX9	0.6765	0.8992	ATP-dependent RNA helicase A
EIF3A	0.6117	0.8992	Eukaryotic translation initiation factor 3 subunit A
**EIF3C**	0.0548	0.3380	Eukaryotic translation initiation factor 3 subunit C
EIF3E	0.8292	0.9114	Eukaryotic translation initiation factor 3 subunit E
EIF3F	0.9687	0.9702	Eukaryotic translation initiation factor 3 subunit F
EIF3L	0.6828	0.8992	Eukaryotic translation initiation factor 3 subunit L
EIF4E	0.6118	0.8992	Eukaryotic translation initiation factor 4E
ENO2	0.2948	0.6335	Gamma-enolase;Enolase
ESD	0.6537	0.8992	S-formylglutathione hydrolase
**FARSA**	0.0445	0.3380	Phenylalanine-tRNA ligase alpha subunit
FARSB	0.8027	0.9114	Phenylalanine-tRNA ligase beta subunit
FMNL2	0.0497	0.3380	Formin-like protein 2
GNB2L1	0.6204	0.8992	Guanine nucleotide-binding protein subunit beta-2-like 1
HNRNPK	0.5817	0.8992	Heterogeneous nuclear ribonucleoprotein K
HNRNPL	0.9667	0.9702	Heterogeneous nuclear ribonucleoprotein L
ITCH	0.07381	0.3629	E3 ubiquitin-protein ligase Itchy homolog
MCM3	0.7612	0.9114	DNA replication licensing factor MCM3
NAA15	0.2761	0.6335	N-alpha-acetyltransferase 15, NatA auxiliary subunit
NAGK	0.7243	0.9092	N-acetyl-D-glucosamine kinase
NAMPT	0.1475	0.5408	Nicotinamide phosphoribosyltransferase
NANS	0.3736	0.6755	Sialic acid synthase
NARS.1	0.3114	0.6335	Asparagine-tRNA ligase, cytoplasmic
NMT1	0.184	0.5408	Glycylpeptide N-tetradecanoyltransferase 1
NONO	0.174	0.5408	Non-POU domain-containing octamer-binding protein
PLOD1	0.0576	0.3380	Procollagen-lysine,2-oxoglutarate 5-dioxygenase 1
PRKDC	0.1925	0.5408	DNA-dependent protein kinase catalytic subunit
PRMT1	0.4007	0.6755	Protein arginine N-methyltransferase 1
**PRMT5**	0.0490	0.3380	Protein arginine N-methyltransferase 5
PYGL	0.9702	0.9702	Glycogen phosphorylase, liver form;Alpha-1,4 glucan phosphorylase
QDPR	0.2861	0.6335	Dihydropteridine reductase
RAB13	0.3945	0.6755	Ras-related protein Rab-13
RAN	0.2464	0.6321	GTP-binding nuclear protein Ran
RBBP4	0.8342	0.9114	Histone-binding protein RBBP4
RBBP7	0.1015	0.4607	Histone-binding protein RBBP7
RCC2	0.3045	0.6335	Protein RCC2
RPSA	0.7558	0.9114	40S ribosomal protein SA
RTCB	not found		tRNA-splicing ligase RtcB homolog
SDC4	0.0389	0.3380	Syndecan-4
SF3A3	0.9112	0.9698	Splicing factor 3A subunit 3
SF3B3	0.3236	0.6364	Splicing factor 3B subunit 3
SND1	0.6355	0.8992	Staphylococcal nuclease domain-containing protein 1
UBA1	0.5517	0.8992	Ubiquitin-like modifier-activating enzyme 1
**VARS**	0.0165	0.3380	Valine-tRNA ligase
VPS45	0.2086	0.5594	Vacuolar protein sorting-associated protein 45
WDR61	0.8338	0.9114	WD repeat-containing protein 61
XRCC5	0.3829	0.6755	X-ray repair cross-complementing protein 5
XRCC6	0.719	0.9092	X-ray repair cross-complementing protein 6

Table S2

		hEV A375					hEV MelJuso					hEV IPC298					hEV 501Mel				
		Technical replicates		Biological replicates			Technical replicates		Biological replicates			Technical replicates		Biological replicates			Technical replicates		Biological replicates		
		QPCR Array	1st	2nd	3rd	4th	QPCR Array	1st	2nd	3rd	4th			1st	2nd	3rd			1st	2nd	3rd
**Target**		**Fold change**					**Fold change**					**Fold change**					**Fold change**				
miR-210	**Up under hypoxia**	13,9	6,5	4,1	8,3	3,5	13,6	6,8	8,1	4,1	20,9			3,9	3,1	3,8			5,3	6,4	4,1
hsa-miR-1290		30,9	29,8	5,4	2,0	2,0	4,0	3,2	7,1	3,3	11,9			11,9	6,5	9,6			8,4	7,1	13,8
hsa-miR-323a-5p		3,8	3,3	1,3	0,9	0,8	2,8	2,3	2,0	1,0	4,4			7,1	8,0	5,7			2,2	2,5	4,0
hsa-miR-23a-5p	**Down under hypoxia**	0,1	0,1	0,4	0,6	0,3	0,1	0,4	0,2	0,2	0,3			0,5	0,2	0,3			0,6	0,3	0,3
hsa-miR-23b-5p		0,1	0,1	0,3	0,4	0,5	0,2	0,1	0,3	0,1	0,1			0,9	0,3	N/A			0,5	0,4	0,3
hsa-miR-708-5p		0,2	0,4	0,6	0,8	1,0	0,9	0,7	0,8	0,7	0,4			0,6	0,4	0,3			0,5	0,4	0,3
Let-7d-3p		0,1	0,1	0,3	0,3	0,6	0,4	0,4	0,1	0,7	0,8			0,5	0,4	0,5			0,5	0,3	0,4

Table S2. qPCR validation of selected miRNAs, up-regulated (miR-210, miR-1290, miR-323a-5p) or down-regulated (let-7d-3p, miR-23b-5p, miR-708-5p, miR-23a-5p) for the 4 melanoma cell lines (A375, 501Mel, MelJuso and IPC298). Fold change were calculated by dividing the normalized relative amount of hypoxic sample in the normalized relative amount of normoxic sample. The fold change values are shown for the qPCR array, the technical replicates of the qPCR array and validations in three biological replicates.

Western blots information

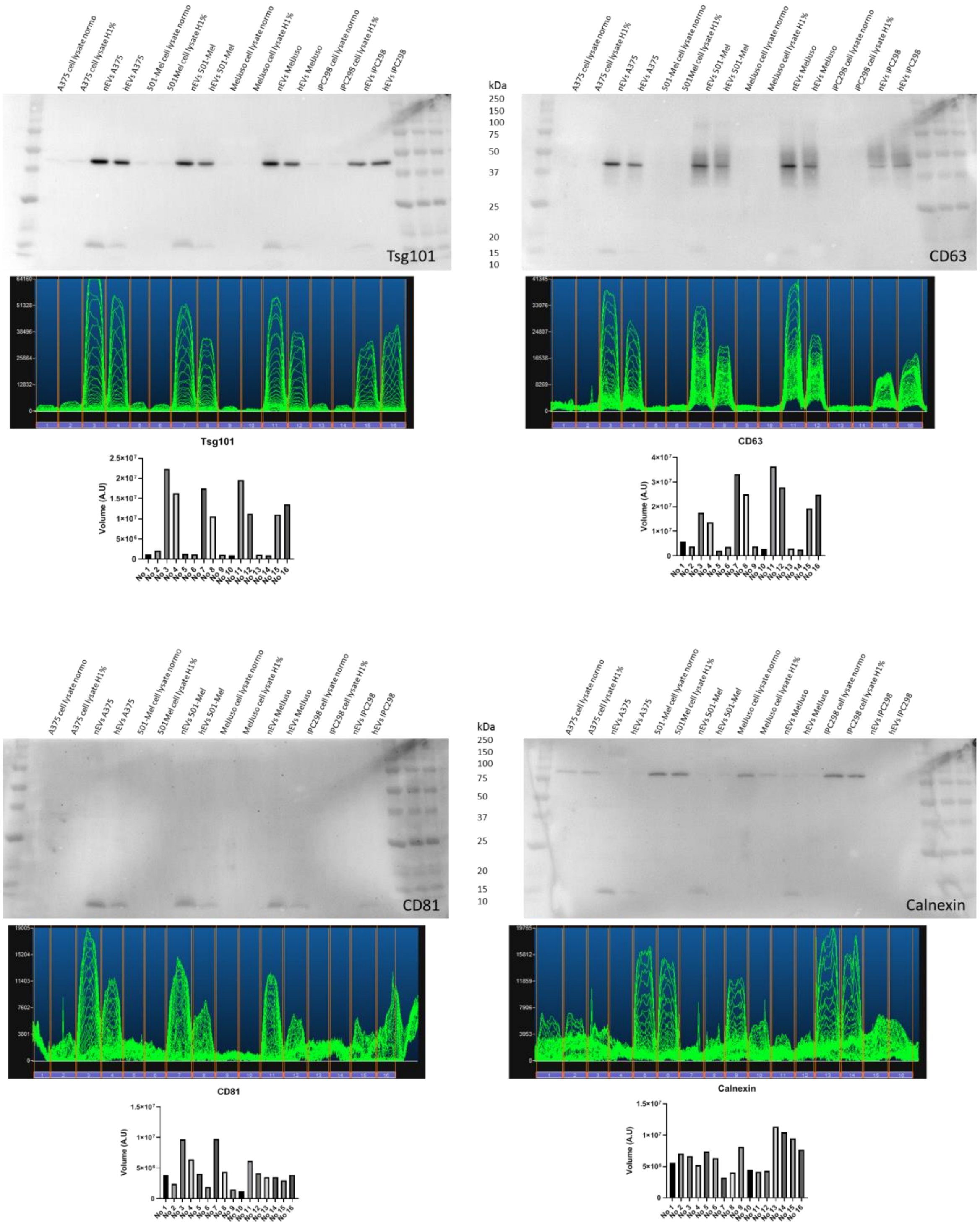

Whole blots and densitometries reading using Vilber Lourmat software of the Western blot Figure 2A.

Western blots information

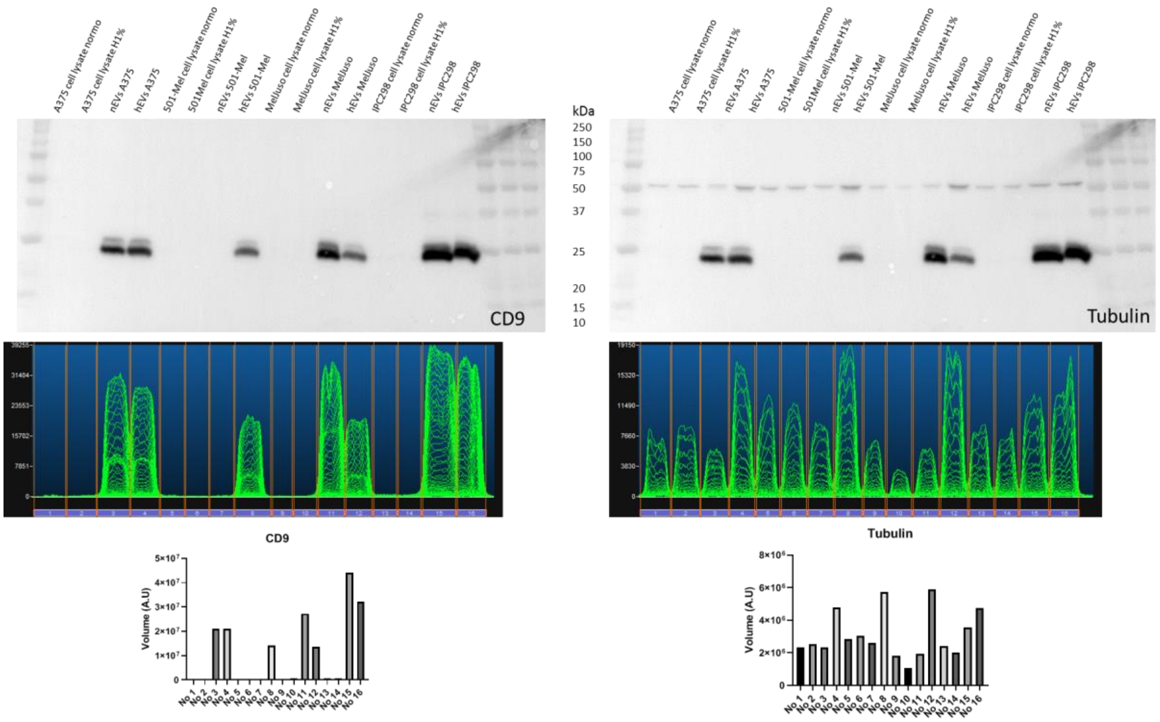

Whole blots and densitometries reading using Vilber Lourmat software of the Western blot Figure 2A.

Western blots information

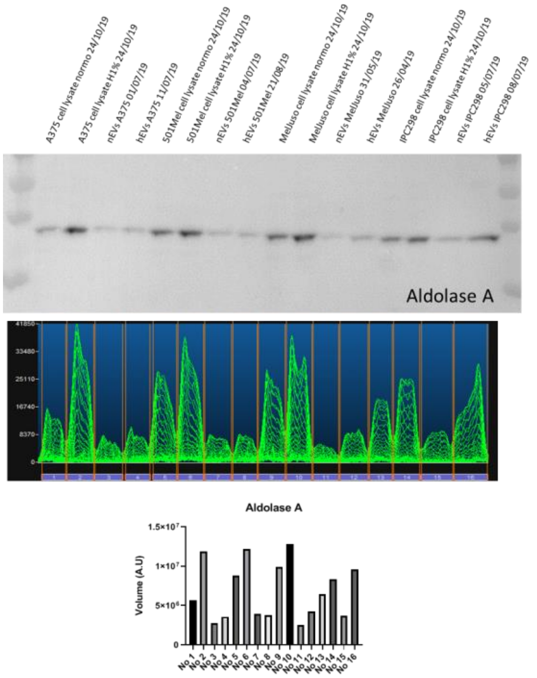

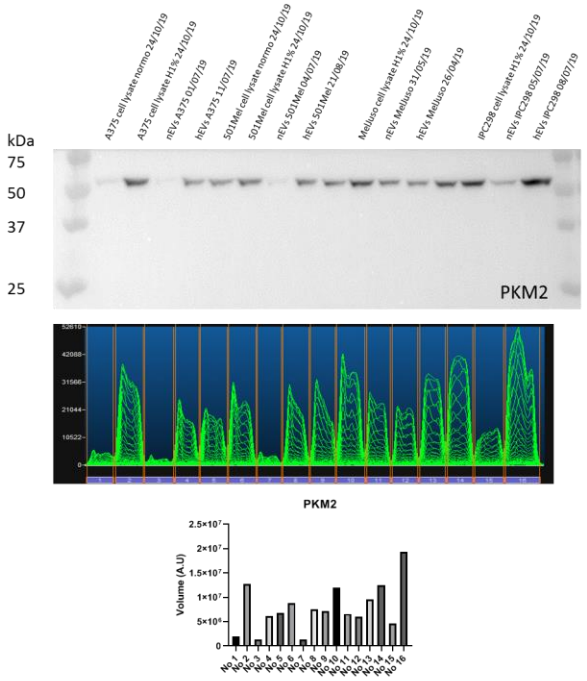

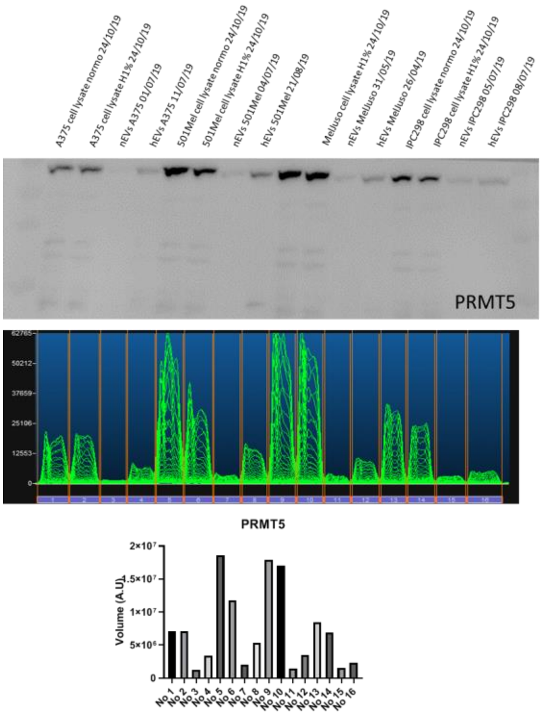

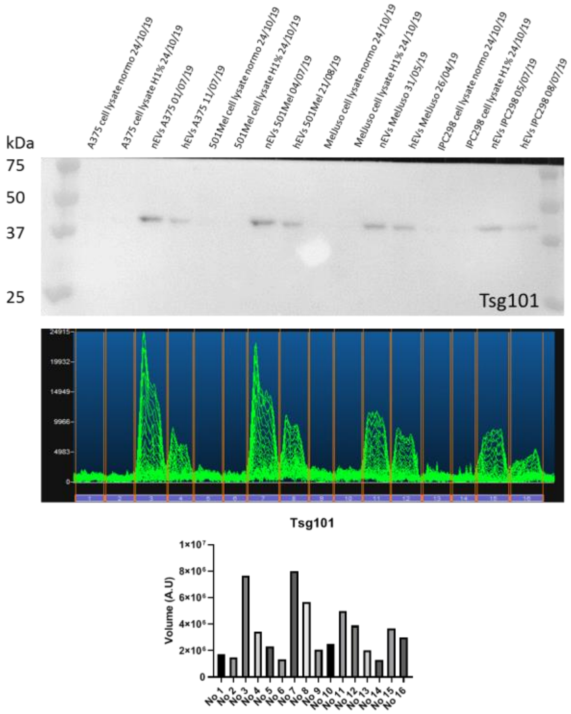

Whole blots and densitometries reading using Vilber Lourmat software of the Western blot Figure 3C.

Western blots information

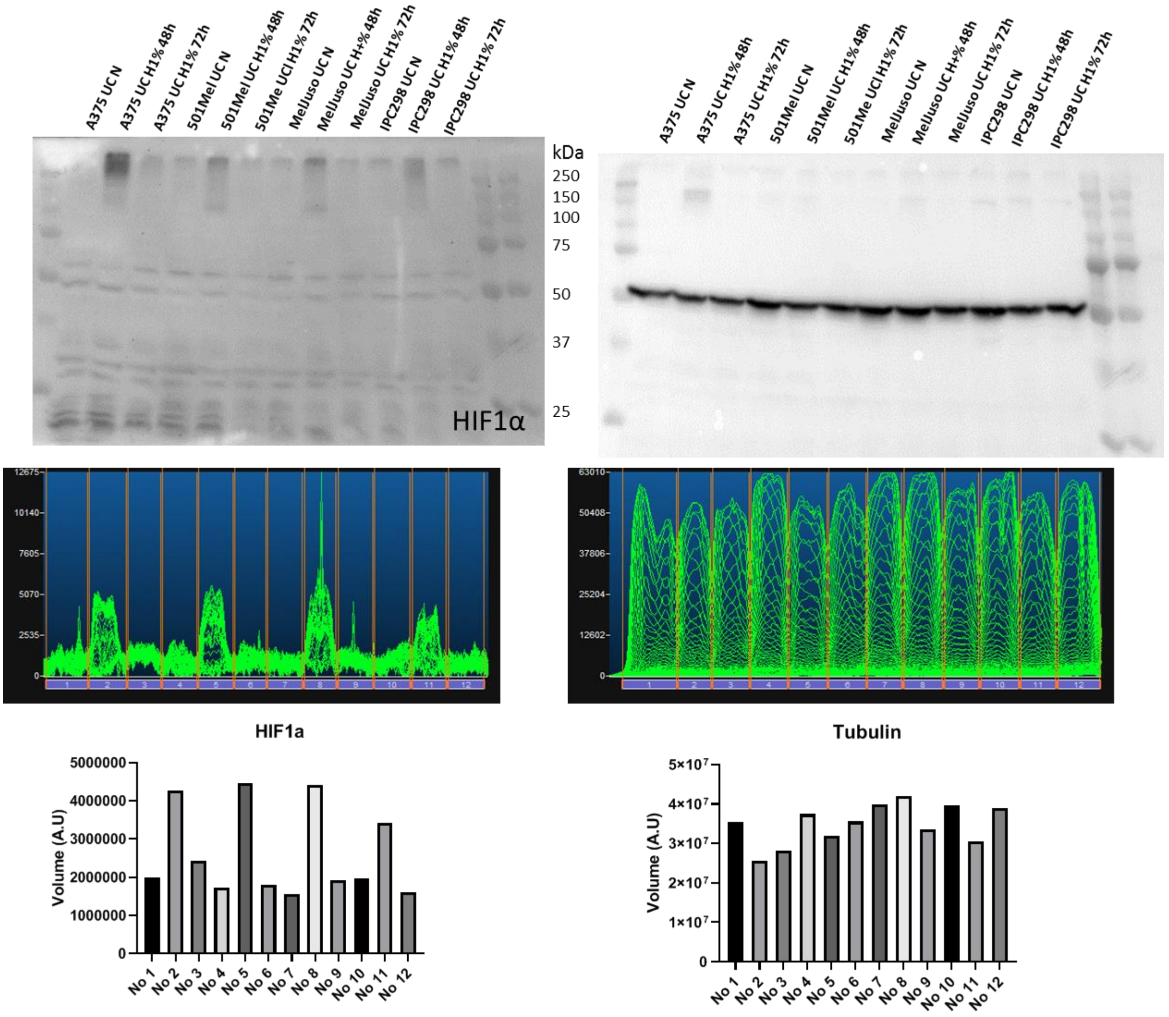

Whole blots and densitometries reading using Vilber Lourmat software of the Western blot Figure S1.
